# Long noncoding RNA gastric cancer-related lncRNA1 mediates gastric malignancy through miRNA-885-3p and cyclin-dependent kinase 4

**DOI:** 10.1038/s41419-018-0643-5

**Published:** 2018-05-22

**Authors:** Zhijuan Lin, Zhixia Zhou, Hang Guo, Yuqi He, Xin Pang, Xumei Zhang, Ying Liu, Xiang Ao, Peifeng Li, Jianxun Wang

**Affiliations:** 1 Center for Tumor Molecular Biology, Institute for Translational Medicine, Qingdao University, Qingdao, 266021 China; 2 Key Lab for Immunology in Universities of Shandong Province, School of Clinical Medicine, Weifang Medical University, Weifang, 261053 China; 3 Department of Anesthesiology, PLA Army General Hospital, Beijing, 100700 China; 4 Department of Gastroenterology, PLA Army General Hospital, Beijing, 100700 China; 5 Department of Pathology, Affiliated Hospital of Weifang Medical University, Weifang, 261041 China

## Abstract

Gastric cancer (GC) is one of the most common malignancy and the third leading cancer-related death in China. Long noncoding RNAs (lncRNAs) have been implicated in numerous tumors, including GC, however, the mechanism of many functional lncRNAs is still unclear. In this study, we identified the abundantly expressed lncRNA, RP11-290F20.3, in GC cells and patient tumor tissues. We named this lncRNA as GC-related lncRNA1 (GCRL1), which could regulate gastric cell proliferation and metastasis, both in vitro and in vivo. Mechanistically, miRNA-885-3p (miR-885-3p) could inhibit the cell proliferation and metastasis in GC by negatively regulating the expression of cyclin-dependent kinase 4 (CDK4) at the post-transcriptional level. Further, GCRL1 promoted the cell proliferation and metastasis by sponging miR-885-3p and hence, positively regulating CDK4 in GC cells. Taken together, our results demonstrate a novel regulatory axis of malignant cell proliferation and invasion in GC, comprising GCRL1, miR-885-3p, and CDK4, which may serve as a potential therapeutic target in GC.

## Introduction

Gastric cancer (GC) is a common malignancy worldwide and one of the top leading causes of cancer mortality in China^1,2^. Its molecular mechanisms are very complicated and still poorly understood^3,4^. Many patients are being diagnosed at an advanced stage so they have to accept extended radical resection of cancer tissues, combined with chemotherapy or radiochemotherapy^5,6^. The 5-year survival rates of < 30% have been reported in patients with advanced GC owing to the high rate of recurrence and metastasis^3,7^. Therefore, it is an urgent clinical need to explore the underlying molecular mechanisms of GC proliferation and metastasis, thus to find specific markers or to set up precise and less harmful strategies for this disease.

Noncoding RNAs (ncRNAs), with microRNAs (miRNAs) and long ncRNAs (lncRNAs) included, which account for about 98% of the genome, have been discovered to take part in the regulation of protein-coding genes in both physiological and in pathological conditions^8–11^. Among them, some miRNAs are reported to be involved in the modulation of the biological behaviors of tumor cells such as cell growth, invasion, autophagy, and apoptosis^12–14^. For example, miR-29c is reported to be one of the lowest expressed miRNAs in GC tissues and could suppress cancer cell migration and induce apoptosis by directly targeting integrin β1 (ITGB1)^14^. LncRNAs are transcripts usually longer than 200 nucleotides (ntds) with limited protein-coding capability. Several lncRNAs such as KRTAP5-AS1^15^, nuclear factor-κB-interacting lncRNA^16^, PNUTS^17^, gallbladder cancer-associated suppressor of pyruvate carboxylase GCASPC^18^, and metastasis-associated lung adenocarcinoma transcript 1 (MALAT1)^19^ have been validated recently as competing endogenous RNAs (ceRNAs) of miRNAs or mRNAs, and regulate gene expression in multiple cancers, including GC. For instance, miR-23b-3p, although could inhibit autophagy by direct binding to autophagy-related protein 12 (ATG12), could also be regulated by MALAT1 as an endogenous sponge, thus inducing chemoresistance in GC^19,20^. Undoubtedly, lncRNAs and miRNAs have been closely related to the regulatory network of GC and exert their potential roles in its carcinogenesis and progression.

Uncontrolled cell division, a core factor for cancer initiation, is mainly mediated by the imbalance of cell cycle machinery such as activation of cyclins and/or cyclin-dependent kinases (CDKs)^21^. Dysregulated cyclin or CDK activity is involved in almost all types of human cancers^20,22–29^. And the regulatory mechanisms of cyclins or CDKs in cancer oncogenesis and progression are also under exploration. For instance, CDK4 has been listed as the direct target of some miRNAs, including miR-206^25^, miR-483-3p^26^, miR-486-5p^27^, miR-506^28^, and miR-711^29^. Besides, CDK4/E2F1 signal is regulated by MALAT1^20^ in breast cancer, p21 expression is repressed by oncogenic lncRNA FAL1 in ovarian cancer^30^ and p16 (INK4A) expression is regulated by lncRNA MIR31HG to modulate senescence in melanoma^31^. And the inhibition of CDKs by their regulatory ncRNAs, leading to delayed cell proliferation, cell cycle G1/S phase arrest, or enhanced cell apoptosis, further signifies the involvement of miRNAs and/or lncRNAs in cancer progression^20,25–29^. However, molecular mechanisms of CDKs besides cell cycle regulation might exist according to recent researches on CDKs in sarcoma, breast cancer, and GC^32–34^, and need to be clarified. Moreover, the cross talk between CDKs and lncRNAs and/or miRNAs indicates the complexity of the cancer regulatory network, which needs to be explored further.

In our study, the microarray transcriptome analysis was performed for GC-related lncRNA screening with GC tissues and paired normal adjacent gastric tissues. Based on quantitative real-time PCR (qRT-PCR) validation in more tissue samples and GC cell lines, a highly upregulated lncRNA, lnc-RP11-290F20.3 was identified. We named the lncRNA as “GC-related lncRNA1” or “GCRL1”. We observed that GCRL1 could enhance the cellular proliferation, migration, and metastasis in GC cells both in vitro and in vivo. Besides, we demonstrated a novel regulatory axis, comprising GCRL1, miR-885-3p, and CDK4, which is involved in cell proliferation and metastasis in GC. This axis also broadened our understanding of the regulatory mechanism of miRNAs for CDKs in GC. Taken together, our results suggested GCRL1-miR-885-3p-CDK4 as a novel regulatory axis, which may serve as a potential therapeutic target in GC.

## Results

### Upregulation of GCRL1 in GC tissues and cell lines

To identify GC-related lncRNAs, we performed microarray transcriptome analysis to compare lncRNA expression profiles in human GC tissues and their matched adjacent noncancerous tissues. We found that lncRNAs were aberrantly expressed in cancer tissues, compared with those in noncancerous tissues. Furtherly, eight most robustly upregulated lncRNAs were verified by qRT-PCR. Among them, RP11-290F20.3 was significantly upregulated in both GC tissues and cell lines. Although documented in microarray results in GEO (https://www.ncbi.nlm.nih.gov/geo/), no detailed mechanism has been reported about this lncRNA. We chose lnc-RP11-290F20.3 for further research and named it as GCRL1.

**Fig. 1:**
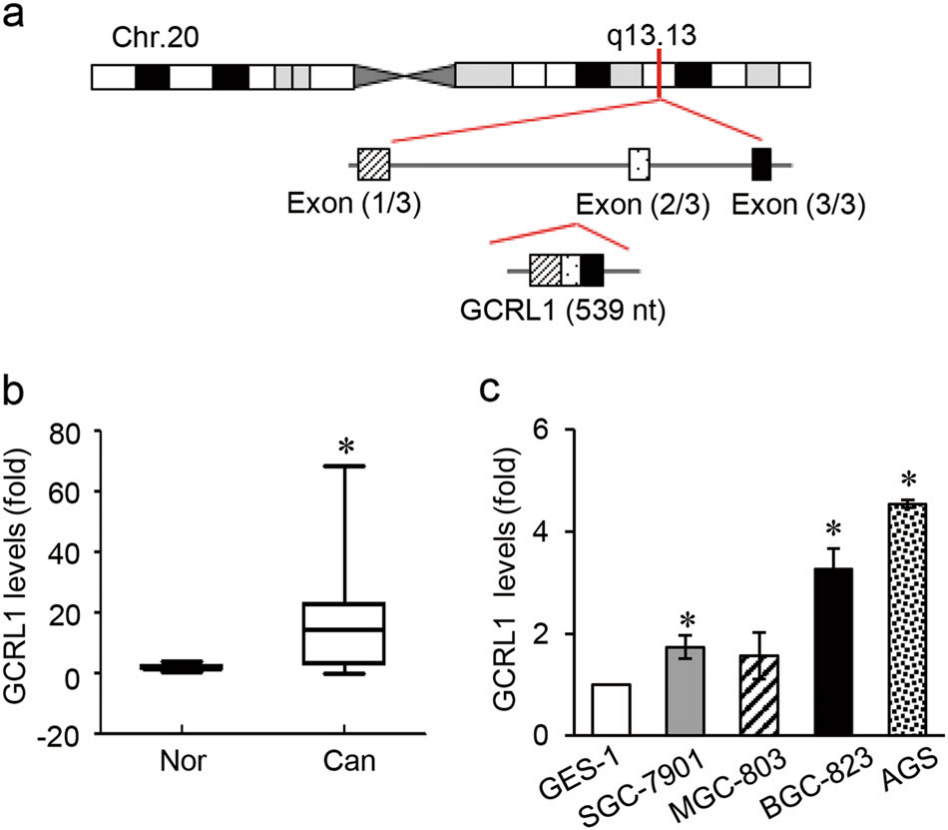
Upregulation of GCRL1 in gastric cancer tissues and cell lines. **a** Schematic map of GCRL1. **b** Relative expression levels of GCRL1 in gastric cancer tissues in comparison with paired adjacent non-tumor tissues (*n* = 26). Data were presented as mean ± SD of fold change, *p < 0.05 compared with non-tumor tissues. **c** Relative expression levels of GCRL1 in gastric cancer cell lines (SGC-7901, MGC-803, BGC-823, and AGS) and normal gastric epithelium cell line GES-1 (*n* = 3). Data are presented as mean ± SD of fold change, *p < 0.05 compared to GES-1 cell. GCRL1 expression was examined by qRT-PCR and normalized to human GAPDH expression (**b**, **c**)

GCRL1 is an intergenic lncRNA located on chromosome 20q13.13, with three exons and a full length of 539 ntds (Fig. 1a). We further validated its expression levels in 26 pairs of human GC tissues and matched adjacent noncancerous tissues by qRT-PCR analysis. The clinicopathological characteristics of the GC patients were shown in Supplementary Table 1. As shown in figure 1b, GCRL1 expression was upregulated in cancerous tissues, with a median difference of approximately 15.9-fold (*p* < 0.05). Moreover, GCRL1 expression levels were also high in SGC-7901, BGC-823, and AGS cells, in comparison to the normal gastric epithelium cell GES-1 (Fig. 1c). Based on these findings, we speculated that GCRL1 might play a vital role in GC development and progression.

### Effects of GCRL1 on GC cell proliferation, migration, and invasion in vitro

**Fig. 2:**
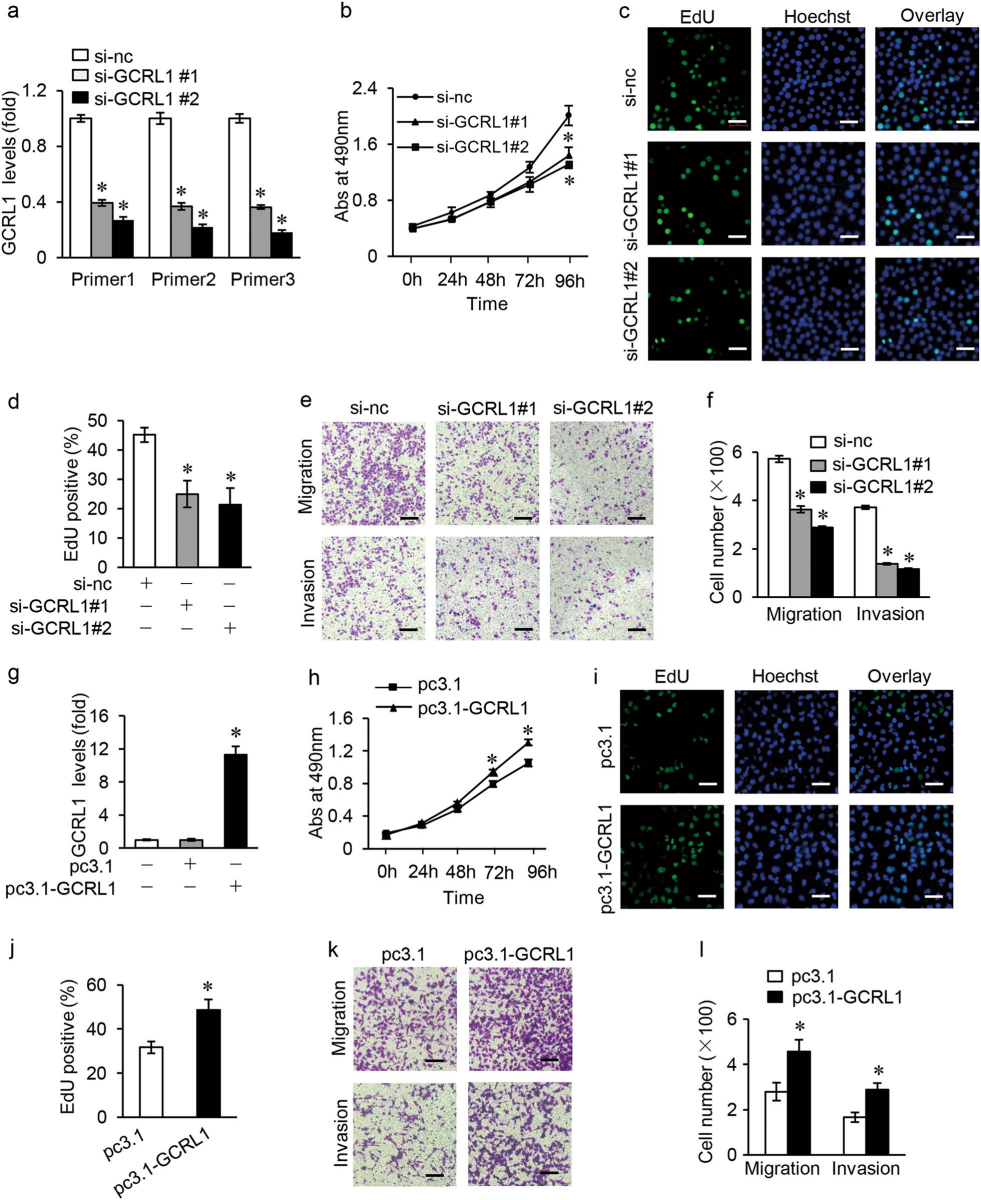
Effects of GCRL1 on gastric cancer cell proliferation, migration, and invasion in vitro. **a**-**f** Loss-of-function assay with BGC-823. **a** Silencing effects of si-GCRL1s examined by qRT-PCR, which were normalized to human GAPDH (*n* = 3). **b** MTT assay showing the cell multiplication of BGC-823 cells treated with si-GCRL1s 24, 48, 72, and 96 h after transfection. Results were shown as absorbance at 490 nm (*n* = 3). **c** EdU incorporation assay indicating the cell proliferation of BGC-823 cells treated with si-GCRL1s and representative images were shown (scale bars = 50 μm). **d** Bar graphs of cell multiplication to **c** upon EdU incorporation assay and results were shown as the ratio of EdU-positive cells to Hoechst-positive cells (*n* = 5). **e** Transwell assay assessing the mobility of BGC-823 cells after GCRL1 knockdown and representative images were shown. (scale bars = 100 μm). **f** Bar graphs of cell mobility to  **e** upon transwell assay and results were shown as the number of migrated or invaded cells per field (*n* = 5). Data are expressed as mean ± SD, *p < 0.05 compared to si-nc group (**a**, **b**, **d**, **f**). **g**-**l** Gain-of-function assay with MGC-803. **g** Enhancements of GCRL1 by pc3.1-GCRL1 examined by qRT-PCR, which were normalized to human GAPDH (*n* = 3). **h** MTT assay showing the cell multiplication of MGC-803 cells with GCRL1 overexpression 24, 48, 72, and 96 h after transfection. Results were shown as absorbance at 490 nm (*n* = 3). **i** EdU incorporation assay indicating the cell proliferation of MGC-803 cells with GCRL1 overexpression and representative images were shown (scale bars = 50 μm). **j** Bar graphs of cell multiplication to  **i** upon EdU incorporation assay and results were shown as the ratio of EdU-positive cells to Hoechst-positive cells (*n* = 5). **k** Transwell assay assessing the mobility of MGC-803 cells with GCRL1 overexpression and representative images were shown (scale bars = 100 μm). **l** Bar graphs of cell mobility to  **k** upon transwell assay and results were shown as the number of migrated or invaded cells per field (*n* = 5). Data are expressed as mean ± SD, *p < 0.05 compared to pc3.1 group (**g**, **h**, **j**, **l**)

BGC-823 cells were used for loss-of-function assays and MGC-803 cells were used for gain-of-function assays. Two small interfering RNAs (siRNAs) targeting GCRL1 were applied to knockdown GCRL1 expression in BGC-823 cells. Three pairs of primers were applied in qRT-PCR to identify the effects of si-GCRL1s, among which primer 1 spanned exon 1 to exon 2, primer 2 spanned exon 2 to exon 3, while primer 3 spanned exon 1 to exon 3 of GCRL1. As shown in figure 2a, both si-GCRL1s could remarkably inhibit the expression of GCRL1. MTT incorporation assay showed that the inhibition of GCRL1 could suppress the proliferation of BGC-823 cells significantly at day 4 (Fig. 2b). The EdU assay further demonstrated that the suppression of GCRL1 attenuated the proliferation of BGC-823 cells (Fig. 2c, d). Moreover, the knockdown of GCRL1 significantly reduced the migrative and invasive cells, as measured by transwell assay (Fig. 2e, f). On the other hand, enforced GCRL1 expression with pcDNA3.1-GCRL1 (pc3.1-GCRL1 for short; Fig. 2g) caused an increased amount of proliferative cells (Fig. 2h-j) and invasive cells (Fig. 2k, l) in MGC-803 cells. However, no change was observed in apoptotic distribution of GCRL1-knockdown BGC-823 cells (Supplementary Fig. S1A-B) or in GCRL1-overexpression MGC-803 cells (Supplementary Fig. S1C-D). Taken together, these data suggest that GCRL1 plays critical oncogenic roles in GC progression.

### Inhibition of GCRL1 suppresses GC proliferation and metastasis in vivo

**Fig. 3:**
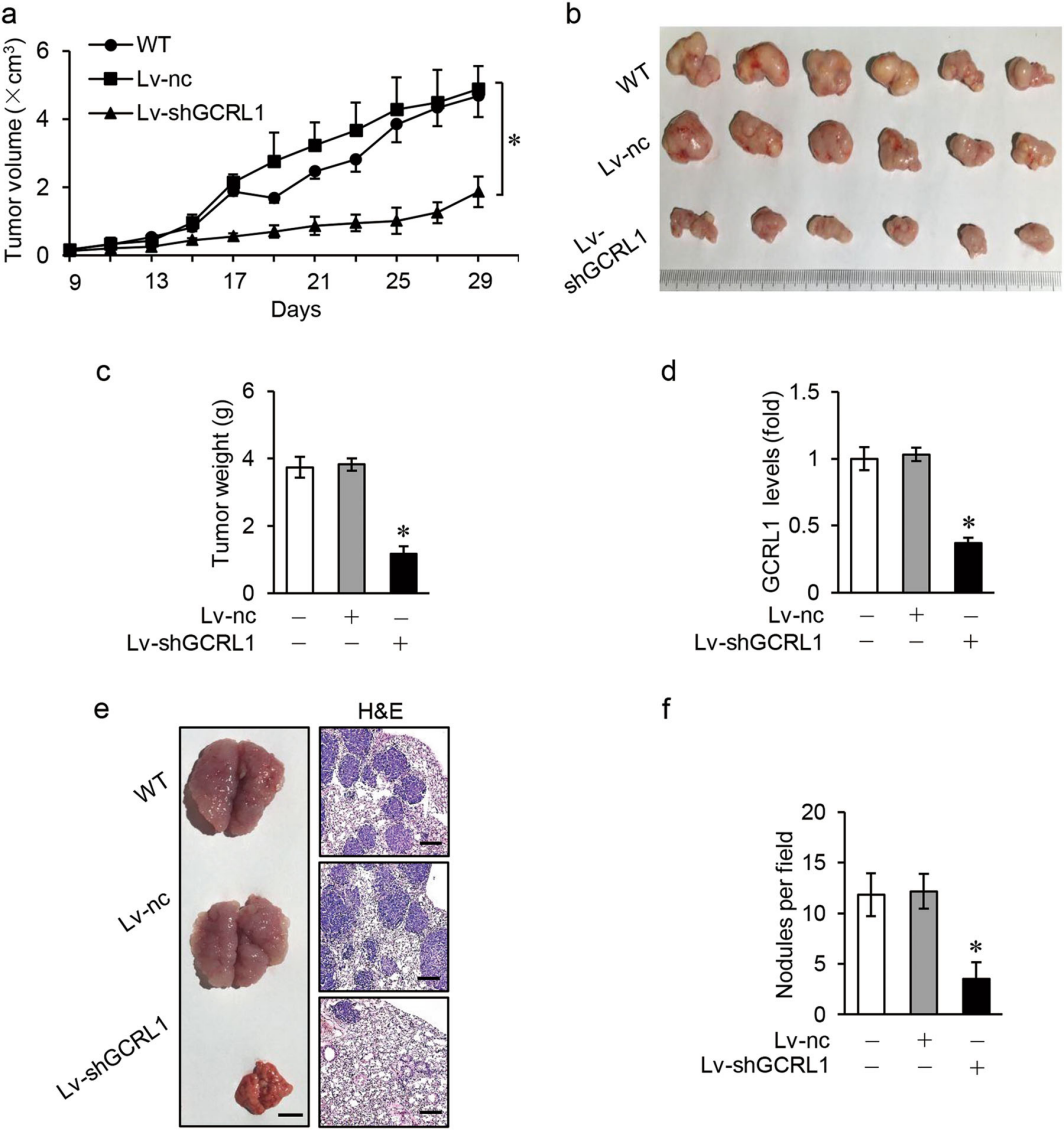
Inhibition of GCRL1 suppresses GC proliferation and metastasis in vivo. **a** Tumor growth curves from day 9 to 29 after subcutaneous injection, with tumor volume calculated as 1/2 × length × width^2^ cm^3^. **b** Morphology of mice tumors dissected at day 29 after injection, *n* = 6 each group. **c** Tumor weight at day 29, *n* = 6 each group. **d** Expression levels of GCRL1 in mice tumor tissues detected by qRT-PCR, normalized to human GAPDH, *n* = 6 each group. **e** Left: photographs showing typical morphology of mice lungs dissected 29 days after tail vein injection (scale bars = 500 μm, *n* = 6 each group). Right: visualization of the dissected mice lungs with H&E staining and representative images were shown (scale bars = 100 μm). (**f**) Bar graphs showing the number of lung metastatic nodules per view, *n* = 6. Data are expressed as mean ± SD, *p < 0.05 compared to Lv-nc group (**a**, **c**, **d**, **f**)

We next analyzed the roles of GCRL1 in vivo, basing on its observed proliferation and invasion promoting effects in vitro. Consistent with the higher knockdown efficiency of si-GCRL1 #2 in vitro, lentivirus based on Lv-shGCRL1 #2 showed a higher silencing effect on GCRL1 confirmed by qRT-PCR (Supplementary Fig. S2A-B) and so Lv-shGCRL1 #2 (Lv-shGCRL1 for short) was used for the following in vivo assays. Mice xenograft model was constructed, monitored, and animals were euthanized 29 days after subcutaneous injection (details described in methods). As shown in figure 3a-c, inhibition of GCRL1 significantly induced a delayed growth of tumor, a reduction in tumor size, and weight in GCRL1 knocked-down group than those in control group. Further detecting GCRL1 in mice tumor tissues, reduced expression level of GCRL1 in GCRL1 knocked-down group than in control group was observed (Fig. 3d).

Meanwhile, lung metastasis models were set up by tail vein injection of BGC-823 cells. When the mice lungs were dissociated at day 29 after injection, we found that the lungs of GCRL1 knocked-down group had a relatively normal size and morphology, while lungs of control mice became much larger and totally solid (Fig. 3e, left). Further hematoxylin-eosin (HE) staining of lung sections indicated that the size and number of lung metastatic nodules in GCRL1 knocked-down group were significantly decreased than that in control group (Fig. 3e, right, Fig. 3f). Collectively, these data suggest that GCRL1 is involved in the proliferation and metastasis of GC.

### GCRL1 directly regulates the expression of miRNA-885-3p

**Fig. 4:**
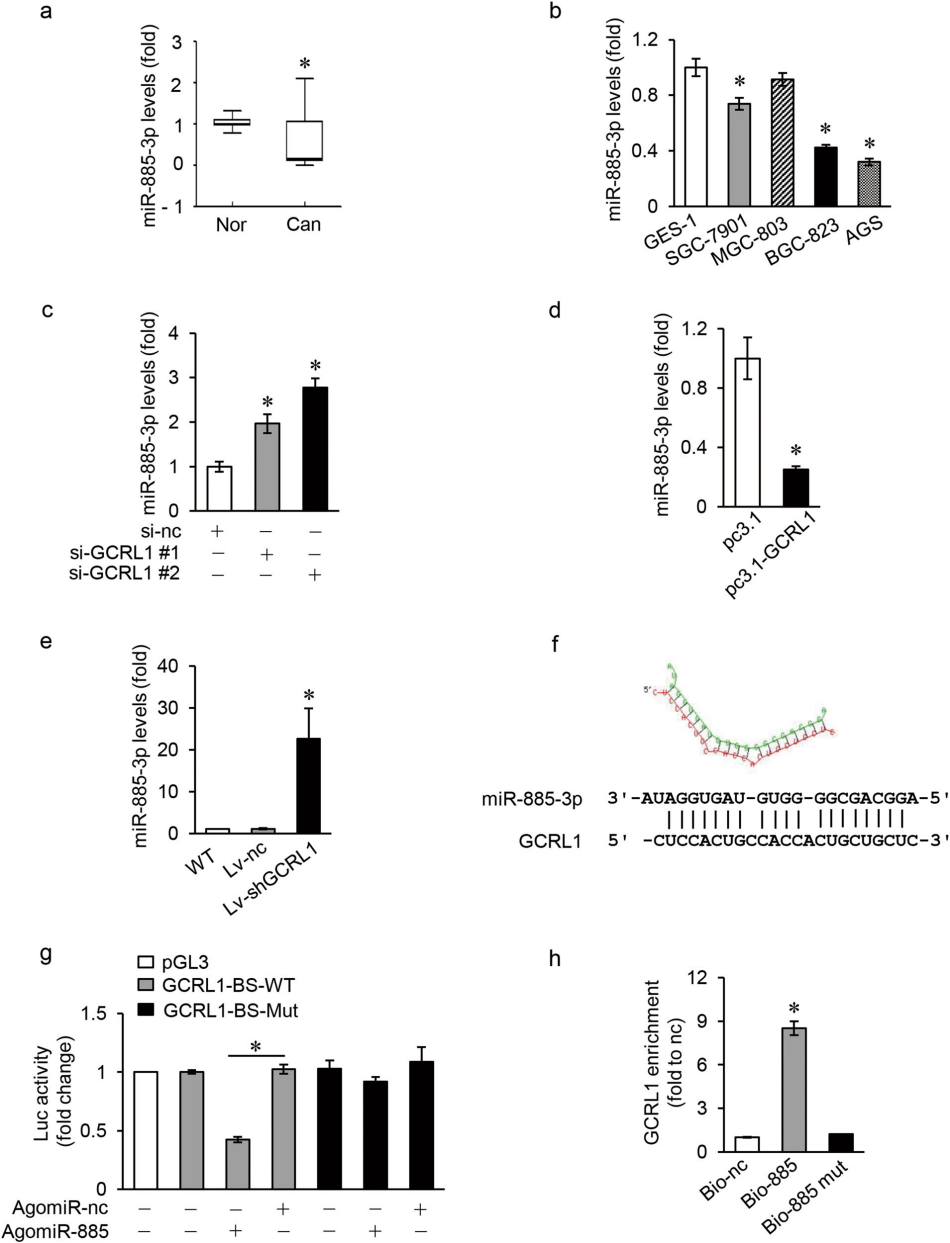
GCRL1 directly regulates the expression of miR-885-3p. **a** Expression levels of miR-885-3p in GC tissues, *n* = 26. **b** Expression levels of miR-885-3p in gastric cancer cell lines (SGC-7901, MGC-803, BGC-823, and AGS) and normal gastric epithelium cell line GES-1 (*n* = 3). **c** Expression levels of miR-885-3p in BGC-823 cells transfected with si-GCRL1s (*n* = 3). **d** Expression levels of miR-885-3p in MGC-803 cells with GCRL1 overexpression (*n* = 3). **e** Expression levels of miR-885-3p in mice tumor tissues, *n* = 6 in each group. **f** Prediction of the binding sites between GCRL1 and miR-885-3p by RNAhybrid. **g** Dual luciferase reporter assay assessing the direct binding between GCRL1 and miR-885-3p in HEK-293T cells (*n* = 3). **h** Detection of GCRL1 by qRT-PCR in BGC-823 cells transfected with Bio-885, Bio-885 mut, or Bio-nc by RNA pull-down assay and results were normalized to human GAPDH (*n* = 3). Data are expressed as mean ± SD, *p < 0.05 compared to adjacent non-tumor tissues (**a**), GES-1 (**b**), si-nc (**c**), pc3.1 (**d**), Lv-nc (**e**), AgomiR-nc (**g**), or Bio-nc (**h**). The detections were performed by qRT-PCR and normalized to human U6 (**a-e**)

One of the mechanisms that lncRNAs function through is to play regulatory roles by acting as ceRNAs for specific miRNAs, then targeting other terminal mRNAs^15–20^. So we set out to find out whether GCRL1 could act as a molecular sponge for miRNAs to regulate the expression of its targets. The interaction probabilities between GCRL1 and miRNAs were predicted by RNAhybrid (http://bibiserv2.cebitec.uni-bielefeld.de/rnahybrid) and DIANA TOOLS (http://diana.imis.athena-innovation.gr/DianaTools/ index. php). miR-185-3p, miR-885-3p, and miR-1250-5p, with the top high mfe values, were predicted to have stronger binding affinities to GCRL1. After validation the expressions of these three miRNAs in GC tissues (Fig. 4a and Supplementary Fig. S3A) and cell lines (Fig. 4b and Supplementary Fig. S3B), we found that miR-885-3p was the most differentially expressed one. Moreover, the knockdown of GCRL1 significantly increased miR-885-3p level in BGC-823 cells (Fig. 4c), while enforced GCRL1 expression markedly reduced miR-885-3p level in MGC-803 cells (Fig. 4d). Further, enhanced expression levels of miR-885-3p could also be observed in mice tumor tissues of Lv-shGCRL1 group compared to that in control groups (Fig. 4e). Taken together, these results suggest that GCRL1 could regulate miRNA-885-3p expression in GC.

The predicted binding sites between miR-885-3p and GCRL1 were shown in figure 4f. To identify the direct binding between GCRL1 and miR-885-3p, the wild type and mutant GCRL1 fragment containing miR-885-3p-binding site were synthesized and cloned into downstream of the luciferase reporter gene (GCRL1-BS-WT and GCRL1-BS-Mut) (Supplementary Fig. S4). After co-transfection of GCRL1-BS-WT or GCRL1-BS-Mut together with miR-885-3p, luciferase activity was analyzed. As shown in figure 4g, GCRL1-BS-WT could decrease the luciferase activity about 50% in HEK-293T cells while GCRL1-BS-Mut could not. Furthermore, biotinylated miR-885-3p (Bio-885), miR-885-3p mutant (Bio-885 mut), or RNA control (Bio-nc) was transfected into BGC-823 cells for GCRL1 pull-down assays. As shown in figure 4h, GCRL1 could only be enriched significantly in cells transfected by Bio-885. These results indicate that GCRL1 could bind to miR-885-3p directly and plays a negative regulatory role for miR-885-3p expression.

### miR-885-3p suppresses GC cells proliferation and metastasis in vitro and in vivo

**Fig. 5:**
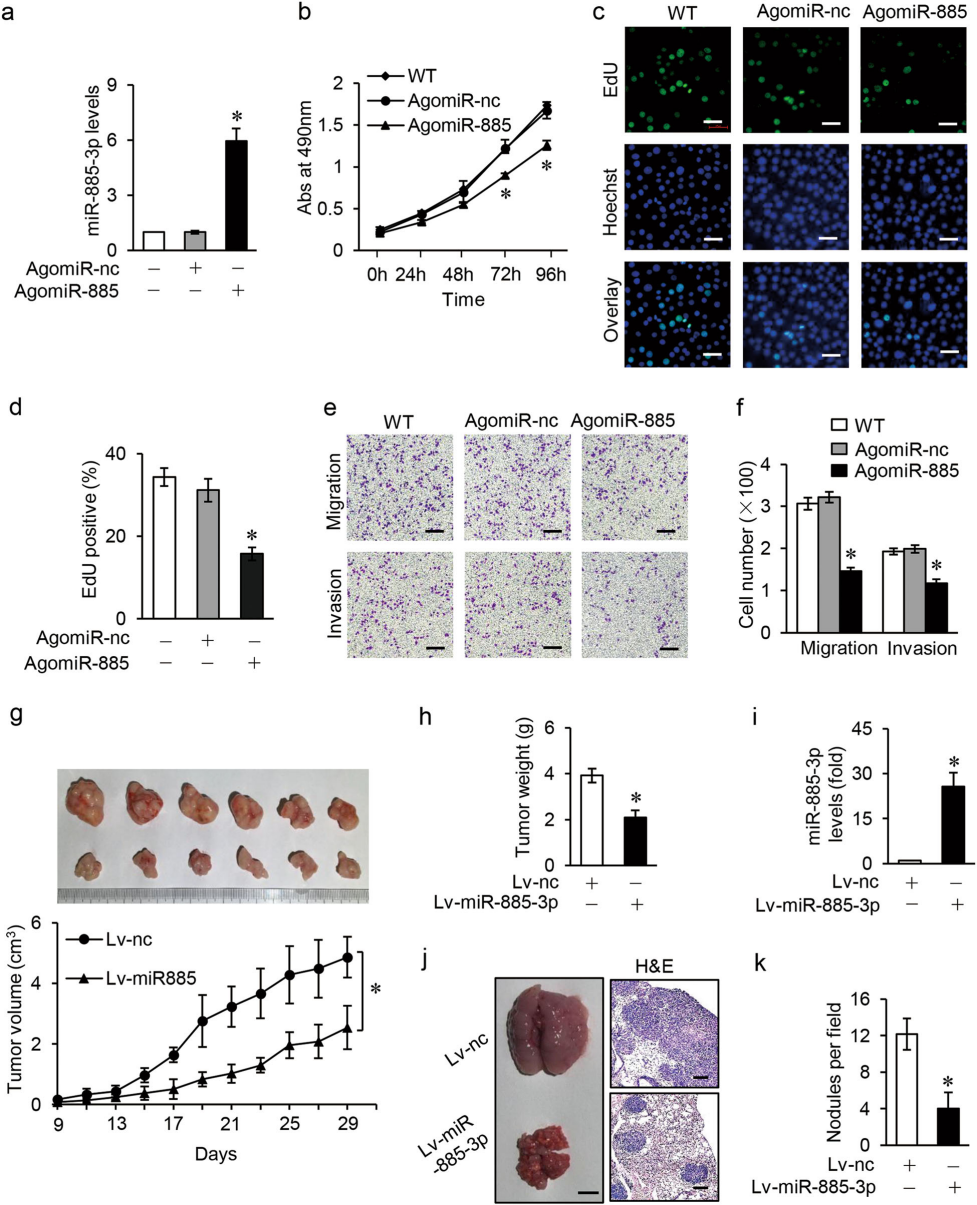
miR-885-3p suppresses GC proliferation and metastasis in vitro and in vivo. **a** Expression levels of miR-885-3p determined by qRT-PCR in BGC-823 cells transfected with AgomiR-885, which were normalized to human U6 (*n* = 3). **b** MTT assay showing the cell multiplication of BGC-823 cells treated with AgomiR-885 24, 48, 72, and 96 h after transfection. Results were shown as absorbance at 490 nm (*n* = 3). **c** EdU incorporation assay indicating the cell proliferation of BGC-823 cells treated with agomiR-885 and representative images were shown (scale bars = 50 μm). **d** Bar graphs of cell multiplication to  **c** upon EdU incorporation assay and results were shown as the ratio of EdU-positive cells to Hoechst-positive cells (*n* = 5). **e** Transwell assay assessing the mobility of BGC-823 cells with miR-885-3p enhancement by AgomiR-885 and representative images were shown (scale bars = 100 μm). (**f**) Bar graphs of cell mobility to  **e** upon transwell assay and results were shown as the number of migrated or invaded cells per field (*n* = 5). **g** Up: morphology of mice tumors dissected at day 29 after injection, *n* = 6 in each group. Down: tumor growth curves from day 9 to 29 after subcutaneous injection, with tumor volume calculated as 1/2 × length × width^2^ cm^3^. **h** Tumor weight at day 29, *n* = 6 each group. **i** Expression levels of miR-885-3p in mice tumor tissues detected by qRT-PCR, normalized to human U6, *n* = 6 each group. **j** Left: photographs showing typical morphology of mice lungs dissected 29 days after tail vein injection (scale bars = 500 μm, *n* = 6 each group). Right: visualization of the dissected mice lungs with H&E staining and representative images were shown (scale bars = 100 μm). **k** Bar graphs showing the number of lung metastatic nodules per view, *n* = 6. Data are expressed as mean ± SD, *p < 0.05 compared to AgomiR-nc (**a**, **b**, **d**, **f**) or Lv-nc (**g**-**k**)

Till now miR-885-3p has been reported to play different roles in several types of tumors^35–37^, so its effect in GC needs further dissection. miR-885-3p agomiR was used to mock the action of miR-885-3p (Fig. 5a) and miR-885-3p antagomiR was used to knockdown the endogenous miR-885-3p levels (Supplementary Fig. S5A). Accordingly, enforced expression of miR-885-3p inhibited cell proliferation (Fig. 5b-d), migration, and invasion of BGC-823 cells (Fig. 5e, f), whereas knockdown of endogenous miR-885-3p promoted cell proliferation, migration, and metastasis of MGC-803 cells (Supplementary Fig. S5B-E).

Next, we prepared Lv-miR-885-3p to ensure the stable overexpression of miR-885-3p in BGC-823 cells (Supplementary Fig. S6A-B). The athymic nude mice bearing human BGC-823 xenografts were used to investigate the effects of miR-885-3p in vivo. The decreased tumor growth, tumor size, and tumor weight at terminal point on day 29 were observed in Lv-miR-885-3p group compared to Lv-nc control group (Fig. 5g, h). Further detecting miR-885-3p in mice tumor tissues, enhanced expression level of miR-885-3p in GCRL1 knocked-down group than that in control group was observed (Fig. 5i). Moreover, the whole lung of Lv-miR-885-3p group showed a relatively normal appearance and size compared to control group as shown in figure 5j, left, with the latter almost wholly solid. And this difference was furtherly confirmed by hematoxylin and eosin staining of lung sections (Fig. 5j, right). Furthermore, the number of lung metastatic nodules was also reduced in miR-885-3p overexpression mice group (Fig. 5k). Together, these results show that miR-885-3p probably acts as a tumor suppressor in GC.

### miR-885-3p directly targets CDK4

**Fig. 6:**
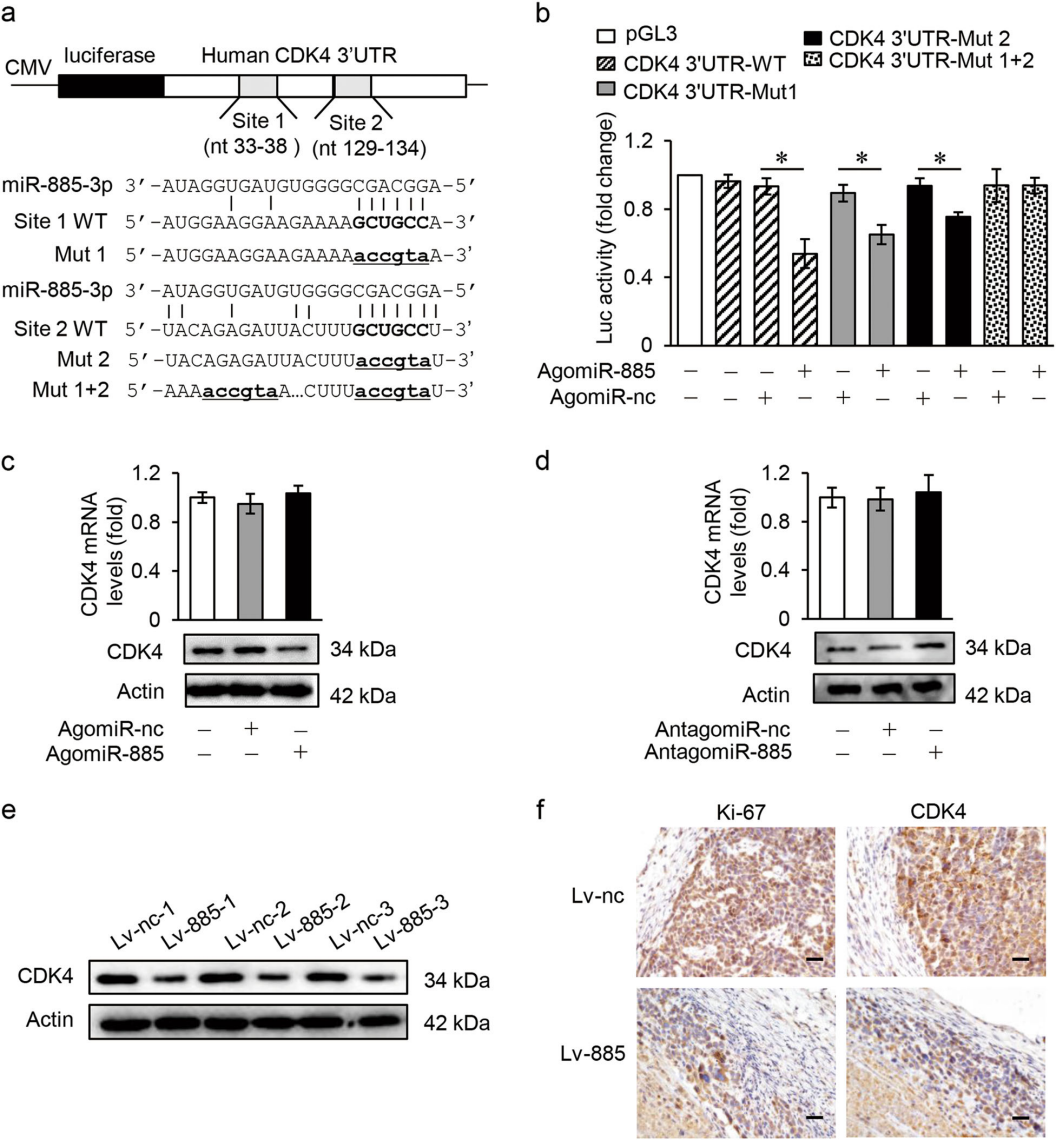
miR-885-3p directly targets CDK4. **a** Up: putative miR-885-3p-binding sites in the 3′-untranslated region (3′UTR) of human CDK4; down: wild type and three mutants of CDK4 3′UTR, with the mutant sites labeled in lowercase letters and underlines. **b** Relative luciferase activities of CDK4 3′UTR reporter in HEK-293T cells (*n* = 3). **c** Expression levels of CDK4 detected by qRT-PCR (up) and western blotting (down) in BGC-823 cells treated with AgomiR-885, normalized to human GAPDH (up) or with β-actin as a control (down) (*n* = 3). **d** Expression levels of CDK4 detected by qRT-PCR (up) and western blotting (down) in MGC-803 cells treated with AntagomiR-885, normalized to human GAPDH (up) or with β-actin as a control (down) (*n* = 3). **e** Expression levels of CDK4 analyzed by western blotting in mice tumor tissues, with β-actin as controls. **f** Expression levels of CDK4 examined by immunohistochemical assay and representative images were shown, with ki-67 as a control (scale bars = 50 μm). Data are expressed as mean ± SD, *p < 0.05 compared to AgomiR-nc (**b**)

To further explore the mechanism that miR-885-3p plays its tumor suppressive role in GC, online resources TargetScan and RNAhybrid were utilized to predict its potential target genes. According to the prediction data, CDK4, which has two separate binding sites, site 1 (nt 33-38) and site 2 (nt 129-134; Fig. 6a, up), has a much higher binding affinity for miR-885-3p in its 3′-untranslated regions (3′UTRs). To find out if CDK4 was a direct target of miR-885-3p, wild-type *CDK4* 3′UTR fragment containing miR-885-3p-binding sites (*CDK4* 3′UTR-WT) and its three mutants, including single site 1-mutant (*CDK4* 3′UTR-Mut 1), single site 2-mutant (*CDK4* 3′UTR-Mut 2), and double site-mutant (*CDK4* 3′UTR-Mut 1 + 2) were synthesized and cloned downstream of the pGL3-control vector (Fig. 6a down). Dual luciferase reporter assay showed that miR-885-3p reduced the luciferase activities significantly in pGL3-*CDK4* 3′UTR-WT-treated HEK-293T cells compared to pGL3-*CDK4* 3′UTR-Mut 1 + 2-treated cells (Fig. 6b). Moreover, miR-885-3p also reduced the luciferase activity in both single site-mutant vector (pGL3-*CDK4* 3′UTR-Mut 1 or pGL3-CDK4 3′UTR-Mut 2)-treated HEK-293T cells (Fig. 6b), confirming the contribution of both potential binding sites for the interaction between miR-885-3p and *CDK4*. These data suggest that miR-885-3p directly binds to *CDK4* 3′UTR through two different binding sites and thus regulate CDK4 expression levels.

As shown in figure 6c, the enforced expression of miR-885-3p reduced the CDK4 expression in BGC-823 cells at the protein levels, but not at the mRNA levels. On the other hand, the inhibition of miR-885-3p promoted CDK4 expression in MGC-803 cells at protein levels not at mRNA levels (Fig. 6d).

As CDK4 expression could be regulated by miR-885-3p in vitro, we next determined its levels in mice tumor tissues. And CDK4 levels were decreased in Lv-miR-885-3p-bearing tumors verified by western blotting (Fig. 6e) and immunohistochemical (IHC) staining of CDK4 (Fig. 6f). Ki-67, a cell proliferation marker, was detected as a positive control. All these data suggest that *CDK4* is one target gene of miR-885-3p and thus might be involved in miR-885-3p-related functions in GC.

### GCRL1 regulates the proliferation and metastasis of GC via miR-885-3p and CDK4

CDK4, also known as cell division protein kinase 4, is a member of the CDK family that plays essential roles in oncogenesis of various types of tumors. However, specific CDK inhibitors have shown migration and metastasis inhibitory effects especially in sarcoma and advanced breast cancer, indicating CDK functions out of cell cycle controlling^32,33^. So we analyzed the potential roles of CDK4 in GC proliferation and metastasis. The knockdown of CDK4 inhibited the cellular proliferation, migration, and invasion in BGC-823 cells (Supplementary Fig. S7A-D). On the contrary, the overexpression of CDK4 promoted the proliferation, migration, and invasion in MGC-803 cells (Supplementary Fig. S7E-H).

**Fig. 7:**
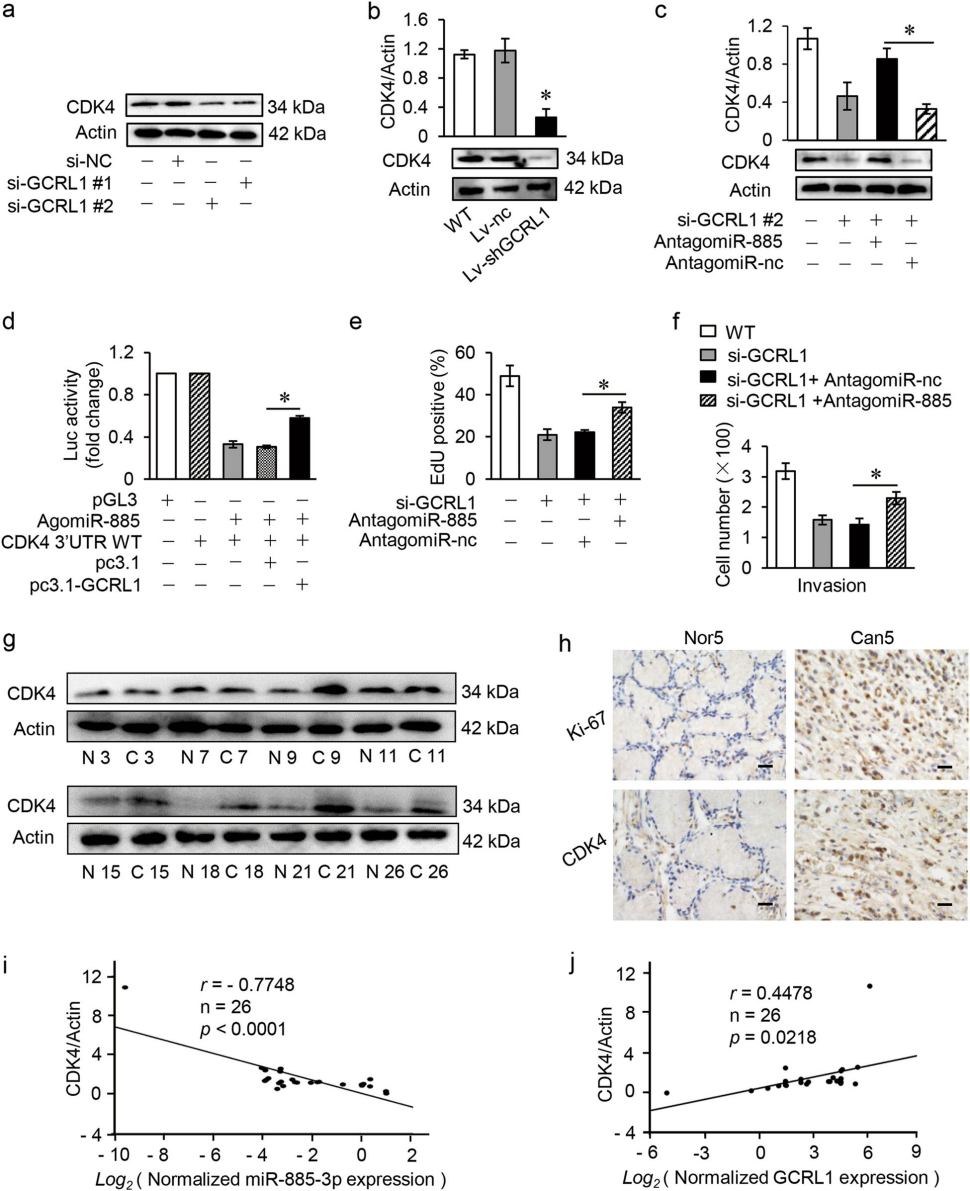
GCRL1 regulates the proliferation and metastasis of GC via miR-885-3p and CDK4. **a** Expression levels of CDK4 detected by western blotting in BGC-823 cells with GCRL1 knockdown (*n* = 3). **b** Expression levels of CDK4 detected by western blotting in mice tumor tissues, quantitated with ImageJ and shown as relative CDK4/Actin expression levels (up) and representative results were shown (down) (*n* = 3). **c** Expression levels of CDK4 detected by western blotting in BGC-823 cells after co-transfection with si-GCRL1 #2 and AntagomiR-nc, or with si-GCRL1 #2 and AntagomiR-885, quantitated with ImageJ and shown as relative CDK4/Actin expression levels (up) and representative results were shown (down) (*n* = 3). **d** Relative luciferase assay assessing the interaction between GCRL1, miR-885-3p, and CDK4 (*n* = 3). **e** Cell proliferation rescue assay was performed in GCRL1-silenced BGC-823 cells transfected with AntagomiR-885 or AntagomiR-nc, and results were shown as the percentage of EdU-positive cells to Hoechst-positive cells (*n* = 3). (**f**) Cell invasion assay was processed in GCRL1-silenced BGC-823 cells transfected with AntagomiR-885 or AntagomiR-nc, and results were shown as the number of invaded cells per field (*n* = 3). **g** Expression levels of CDK4 determined by western blotting in patient tissues of gastric cancer compared to adjacent non-tumor tissues, with β-actin as controls (*n* = 8). **h** Expression levels of CDK4 detected in gastric cancer tissues of patients by immunohistochemical assay and representative images were shown, with ki-67 as controls (scale bars = 50 μm). **i** The association between miR-885-3p and CDK4 protein levels was identified with Pearson correlation analysis, *r* = - 0.7748. **j** The association between GCRL1 and CDK4 protein levels was identified with Pearson correlation analysis, *r* = 0.4478. Data are expressed as mean ± SD, *p < 0.05 compared to Lv-nc (**b**), AntagomiR-nc (**c**, **e**, **f**), and pc3.1 (**d**)

Considering the regulatory relationship between GCRL1-miR-885-3p, and miR-885-3p-CDK4, we hypothesized that GCRL1 might regulate the expression levels and functions of CDK4. We treated BGC-823 cells with si-GCRL1s with or without miR-885-3p blockage and determined the protein levels of CDK4. As shown in figure 7a, the protein levels of CDK4 were decreased due to knockdown of GCRL1 in BGC-823 cells. And CDK4 levels were also reduced in mice tumor tissues bearing BGC-823/Lv-GCRL1 as shown in figure 7b. Next, si-GCRL1-induced decrease of CDK4 could be partially rescued by miR-885-3p antagomiR as shown in figure 7c. Moreover, the dual-luciferase reporter assay (DLR) assay indicated that GCRL1 overexpression could rescue the decreased luciferase activity due to the direct binding of miR-885-3p and CDK4 3′UTR as shown in figure 7d. Further, the decreased proliferation and metastasis of BGC-823 cells by GCRL1 knockdown could also be partly rescued by miR-885-3p antagomiR as shown in figure 7e, f. All these data suggest that GCRL1 could promote cell proliferation and invasion at least partially by positive regulation of CDK4 through sponging miR-885-3p in GC.

To further elucidate the relationship between GCRL1, miR-885-3p, and CDK4, we examined this novel axis in GC tissues of patients. CDK4 is highly expressed in most GC tissues (18/26) compared to adjacent normal tissues and representative protein expression was shown by western blotting analysis (Fig. 7g) and by IHC staining (Fig. 7h). Moreover, the correlation analysis was performed to find out the association between CDK4, GCRL1, and miR-885-3p. With the relative CDK4 protein levels as *x*-axis and log2 of normalized GCRL1 or miR-885-3p expression levels as *y*-axis as shown in figure 7i, j, CDK4 levels were correlated to miR-885-3p negatively (*r* = -0.7748, *p* < 0.0001) and to GCRL1 positively (*r* = 0.4478, *p* = 0.0218) indicated by Pearson correlation analysis in tumor tissues of patients. Our data, therefore, support a model in which the highly expressed GCRL1 promotes cell proliferation and metastasis by positively regulating CDK4 through sponging the miR-885-3p in GC.

## Discussion

LncRNAs have been reported both as tumor suppressors such as GAS5^38^, MEG3^39^, and SPRY4-IT1^40^, and oncogenic such as GClnc1^41^, PVT1^42^, and TUG1^43^ in GC. Here we identify GCRL1 as a highly expressed lncRNA, which promotes cell proliferation and metastasis in GC by sponging miR-885-3p and positively regulating CDK4, member of CDK family. GCRL1 is one of the isoforms of intergenic lncRNA LINC01272, located on chromosome 20q13.13. The lncRNA, LINC01272, has been recently listed as one of the top 10 remarkable biomarkers in lung squamous cell carcinoma (LUSC) based on their highest diagnostic value^44^. Moreover, LINC01272 has been associated to the survival time of LUSC significantly^44^. However, the detailed mechanism for LINC01272 has not been explored yet. This is the first report to provide direct biochemical evidence that GCRL1 sponges miR-885-3p and promotes CDK4 protein levels in GC. The lncRNAs have been reported to play essential roles at multiple levels such as chromatin modification, transcription, and post-transcriptional processing^8–11^. Mechanically, lncRNAs act as scaffolds or guiders to regulate the interactions between genes and proteins^8,41^, as decoys to bind miRNAs or proteins^15,17^, and as enhancers to modulate transcription of terminal or neighboring targets^31,42,45^. Among these mechanisms, lncRNAs as competing endogenous RNAs for miRNAs has been highlighted recently. For instance, a G > A polymorphism (rs11655237) in exon 4 of LINC00673 generates a binding site for miR-1231, and abolishes the inhibitory effect of LINC00673 in an allele-specific manner and thus confers sensitivity to carcinogenesis in pancreatic cancer^46^. Bioactive lncRNA activated in renal clear cell carcinoma (RCC) with Sunitinib resistance (lncARSR) incorporated into exosomes has been reported to induce resistance to Sunitinib when transferred to drug-sensitive cells, mainly by competitively binding to miR-34/miR-449 for enhancing the expression of AXL and c-MET expression in RCC cells^47^. These studies highlight the importance of interaction between lncRNAs and miRNAs in tumorigenesis both as the upstream regulator or the downstream target. Similarly, our results confirmed that GCRL1 could act as upstream regulator for miR-885-3p and promoted proliferation and metastasis in GC cells. Our data show that GCRL1 is abundantly expressed in several GC cell lines and tumor tissues. The knockdown of GCRL1 indeed reduced the cell motility and invasion in vitro and in vivo. As we can see in tumor xenograft models, GCRL1 knockdown significantly reduced the tumorigenesis and metastasis ability of BGC-823 cells in vivo. In addition, the inhibition of GCRL1 induced differential enforcement of miR-885-3p both in BGC-823 cells and in mice tumor tissues with GCRL1 knocking down.

Recently, miR-885-3p has been reported to be involved in the pathogenesis of several types of cancers^35–37,48,49^. For instance, miR-885-3p has been reported as one of the abundantly expressed miRNAs in malignant mesotheliomas, however, the molecular mechanisms need to be explored further^48^. Furthermore, miR-885-3p is reported to be abundantly expressed in many advanced and metastatic tumors. The γ-synuclein, a protein upregulated in several types of cancer, has been shown to upregulate miR-885-3p^49^. On the contrary, miR-885-3p could act as a tumor suppressor both in colon cancer by disrupting angiogenesis via targeting BMPR1A and blocking BMP/Smad/Id1 signaling^35^ and in breast cancer by targeting immunoregulatory protein B7-H3^37^. Our results also demonstrate that miR-885-3p acts as a tumor suppressor in GC. The direct inhibition of miR-885-3p by antagomiR promoted the GC cell proliferation and metastasis in vitro and in vivo. Moreover, inhibition of GCRL1 also differentially increased the levels of miR-885-3p, which could promote cellular proliferation and metastasis by targeting *CDK4* in GC cells both in vitro and in vivo.

CDK4 is a member of CDK family and it has been reported to be upregulated in various tumors. The oncogenic mechanism of CDK4 appears to be primarily focused on cell growth^20,25–29^. However, roles of CDKs in the migration, metastasis, and apoptosis of tumor cells have also been reported^24,32–34^. For instance, cytoplasmic cyclin D1, together with its binding partner CDK4, is reported to regulate cell invasion and metastasis through the phosphorylation of paxillin^24^. CYC065, a CDK2/9 inhibitor, could induce decreased cell migration/invasion and impede the epithelial-mesenchymal transition (EMT) program in triple-negative breast cancer mainly through inhibition of CDK2-mediated phosphorylation of Smad3^33^. Besides, CDK4/6-mediated activation of DUB3 has been reported to be essential to deubiquitinate and stabilize SNAIL1, a key factor promoting EMT and involved in breast cancer metastasis^34^. Consistent with these reports, the expression levels of CDK4 were found to be high in most GC tissues and its silencing inhibited cell proliferation and cell metastasis in BGC-823 cells according to our data. Moreover, the enforced expression of miR-885-3p reduced the CDK4 expression and its inhibition, as expected, promoted CDK4 expression at protein levels by the direct physical interaction to 3′UTR of *CDK4* with its two separate binding sites. Hence, our work verified *CDK4* is one of the target genes of miR-885-3p, which is involved in proliferation and metastasis in GC. Furthermore, the knockdown of GCRL1 decreased CDK4 at protein levels both in vitro and in vivo. Additionally, GCRL1 and CDK4 have shown a positive correlation in most GC tissue samples, however, mechanistically miR-885-3p could only rescue the decreased proliferation and invasion by GCRL1 knockdown to some extent. These results indicate that GCRL1 could regulate CDK4 via miR-885-3p. Because of the limited number of human GC tissues collected in this study, we did not analyze the relevance of GCRL1 or miR-885-3p expression levels to clinicopathologic features and prognosis of these patients. The clinical significance of GCRL1 or miR-885-3p during GC progression needs to be investigated in the further studies.

In summary, we identified that lncRNA, GCRL1, potentially acts as an oncogene in GC. We have provided evidence for the links among GCRL1-miR-885-3p, miR-885-3p-CDK4, and GCRL1-CDK4 in the proliferation and metastasis in GC progression. It remains to be explored whether other miRNAs or mRNAs also participate in the ceRNA network involving lncRNA GCRL1. Our results suggest that GCRL1 depletion may be a promising therapeutic strategy for GC treatment and miR-885-3p may be applied to inhibit tumor progression. Although locked nucleic acid of lncRNAs such as lncARSR has been reported to inhibit the biological functions of lncRNAs or reverse the drug reaction in animals^47^, lncRNAs have not been applied in the clinical till now. Further details about the network of lncRNAs-miRNAs-mRNAs will surely help to fast this process.Taken together, our results demonstrate a novel regulatory axis of cell proliferation and invasion in GC, comprising GCRL1, miR-885-3p, and CDK4, which may serve as a potential therapeutic target in GC.

## Materials and methods

### Tissue samples

A total of 26 fresh GC tissues and their pair-matched normal adjacent gastric tissues were obtained from patients with primary GC. None of these patients has received chemotherapy or radiotherapy. Human tissues were collected during gastroscopy and immediately frozen in liquid nitrogen and stored at −80 °C until used for analysis. Our study protocol was approved by the Research Ethics Committee of Qingdao University and written informed consents were acquired under the agreement of patients with pathological confirmation. Meanwhile, the tumor and patients information were shown in Supplementary Table 1.

### Cell lines

Human GC cell line SGC-7901 was obtained from Institute of Biochemistry and Cell Biology, Chinese Academy of Sciences (Shanghai, China). Human gastric epithelium cell GES-1 and human GC cells MGC-803, BGC-823, and AGS were obtained from Beijing Institute for Cancer Research (Beijing, China) as we described previously^50^. Human embryonic kidney 293T cell (HEK-293T) was purchased from Type Culture Collection of the Chinese Academy of Sciences. All these cell lines were cultured in Dulbecco’s modified Eagle’s medium (Gibco, Grand Island, NY, USA) or RPMI 1640 (Gibco), supplemented with 10% fetal bovine serum, 100 U/ml penicillin, and 100 mg/ml streptomycin (Gibco) in a humidified atmosphere containing 5% CO_2_ at 37 °C. The cells were passaged in our laboratory for fewer than 3 months after receipt. The cells from our frozen stock were used within 5-10 passages and not exceeding a period of 2 months. And their identity was routinely tested.

### RNA extraction and qRT-PCR assays

Total RNA was isolated with TRIzol reagent (Invitrogen) according to the manufacturer’s instructions. Complementary DNA was synthesized with random primers using a reverse transcription kit PrimeScript RT reagent Kit (Takara Biomedical Technology, Dalian, China) or commercial miRNA reverse transcription PCR kit. qRT-PCR analysis was all carried out using the SYBR Premix Ex TaqII kit (Takara Biomedical Technology). The primers for GCRL1*, CDK4*, *GAPDH*, U6, miRNAs (miR-185-3p, miR-885-3p, and miR-1250-5p), and URP, see Supplementary Table 2. All data analysis was operated by real-time PCR system (Biorad Biosystems, Foster City, CA, USA). All results were normalized to the expression of human *GAPDH* or U6.

### siRNAs and miRNA reagents and their application

siRNAs targeting GCRL1 or human *CDK4* were synthesized by RiboBio Co. (Guangzhou, China). And miRNA agomiR or antagomiR was purchased from Genepharma (Shanghai, China). All the siRNAs sequences were shown in Supplementary Table 2. Lipofectamine RNAiMAX (Invitrogen, Carlsbad, CA) was adopted for in vitro transfection of siRNAs and miRNAs. BGC-823 or MGC-803 cells were planted in wells or plates 24 h prior to use with 40–60% confluence, then transfected with siRNAs or miRNA according to the manufacturer’s instructions. The transfected cells were harvested 24 h after transfection for RNA detection and 48 h after transfection for protein detection unless specific notification.

### Plasmid construction and lentivirus preparation

Full length of GCRL1 and *CDK4* were constructed into pcDNA3.1 vector. The shGCRL1 #1- and shGCRL1 #2-targeted GCRL1 were constructed into pLKO.1-shRNA-GFP vector (Addgene, Cambridge, MA), especially for lentivirus package. And pre-miRNA-885-3p was also constructed into this vector for lentivirus package. Co-transfection of pLKO.1-shGCRL1s, pMD2.G and pSpAX2 with lipofectamine 2000 (Invitrogen, Carlsbad, CA, USA) into HEK-293T cells was performed; the supernatants were collected at 48 and 72 h after transfection for lentivirus collection. The efficiency of the lentiviruses was assessed more than 96 h after infection with BGC-823 cell, among which 8 μg/ml polybrene was added to improve the effect of lentiviruses.

### MTT incorporation assay

BGC-823 or MGC-803 cells were seeded onto 96-well plates and cultured overnight. Fresh medium containing MTT (with the final concentration 5 mg/ml) was added to cells 24, 48, 72, and 96 h after siRNA or miRNA agomiR or antagomiR transfection, for further absorbance measurement at 490 nm.

### EdU incorporation assay

EdU incorporation assay was performed to assess the cell proliferation viability according to the instructions provided by the manufacturer (Cell Light EdU DNA imaging Kit, RiboBio, Guangzhou, China). In brief, fresh media with 50 μmol/l EdU was added into proliferating cells 2 h prior to the end of the test for reagent incorporation. After fixation with 4% cold paraformaldehyde for 30 min at room temperature, the cells were washed by three changes of phosphate-buffered saline (PBS), and then neutralized by glycine (2 mg/ml) for 10 min. After washing, the cells were incubated in PBS containing 0.1% Triton X-100 for 30 min. Next, cells were washed again and labeled with 5 μg/ml of Hoechst 33342 for 30 min at room temperature. Images were captured and analyzed with a microscope (Olympus, Tokyo, Japan). The ratio of EdU-stained cells (with green fluorescence) to Hoechst-stained cells (with blue fluorescence) was adopted to examine the cell proliferation activity.

### Apoptosis assay

The cells were harvested and stained with fluorescein isothiocyanate-conjugated Annexin V and the PI cell apoptosis detection Kit (Majorbio Biotech, Shanghai, China) after 24 h transfection according to the manufacturer’s protocol. Results were measured by an FACS Calibur system (BD Biosciences, USA) and analyzed with Flow Jo 7.6.1. All assays were performed in triplicate and representative data were provided.

### Transwell assay

BGC-823 or MGC-803 cells were seeded onto 6-well plates and cultured overnight. After siRNA or miRNA agomiR or antagomiR transfection, cells were collected and counted for further assay. For the invasion assays, we used a 24-well transwell chamber (8 µm, Corning Life Sciences, Corning, New York)) with the upper chamber coated with matrigel (#356234, BD Bioscience). A total of 3 × 10^4^ cells suspended in 100 µl medium containing 1% fetal bovine serum (FBS) were seeded in the top chamber. And 600 µl medium containing 20% FBS was placed to the lower chamber. After incubation for 48 h, cells on the upper membrane surface were softly wiped off using a cotton swab and the lower membrane surface was fixed with ice-cold methanol, stained with 0.5% crystal violet solution, and counted in at least five random fields at the ×100 and ×200 magnification by a microscope (Olympus).

### Luciferase reporter assays

The wild-type fragment or three mutants of human *CDK4* 3′UTR containing putative binding sites for miR-885-3p were synthesized and constructed into the pGL3-control reporter vector (Promega, Madison, WI, USA). The introduction of mutations was listed in figure 6a. HEK-293T cells were harvested 24 h after transfection and the luciferase activity was detected by Dual Luciferase Reporter Assay Kit (Promega) according to the manufacturer’s instructions. Firefly luciferase activities were normalized to Renilla luciferase activity, as described before^51^. All experiments were performed in triplicate.

The similar strategy was performed to assess the regulation relationship between GCRL1 and miR-885-3p. The wild-type and mutant regions of GCRL1 containing putative binding site with miR-885-3p were synthesized. The introduction of mutations was listed in Supplementary Fig. S4.

### Pull-down assay with biotinylated miRNA

BGC-823 cells were transfected with biotinylated miRNA (200 nM), harvested 24 h after transfection. Pull-down assay was performed as formerly described^51^. Briefly, the cells were washed with PBS followed by brief vortex, and incubated in a lysis buffer on ice for 10 min. The lysates were precleared by centrifugation, and 50 μl of the samples were aliquoted for input. The remaining lysates were incubated with streptavidin magnetic beads (Thermo Scientific). After coated with RNase-free bovine serum albumin and yeast tRNA (both from Sigma), the beads were incubated at 4 °C for 3 h, washed twice with ice-cold lysis buffer, three times with the low salt buffer and once with the high salt buffer. The bound RNAs were purified using TRIzol and then for GCRL1 expression analysis by qRT-PCR.

### Western blotting

Anti-CDK4 antibody was purchased from Proteintech (Rosemont, USA) and horseradish peroxidase (HRP)-conjugated Goat anti-Rabbit IgG, HRP-conjugated Goat anti-Mouse IgG, and anti-β-actin antibodies were purchased from Cell Signaling Technology (Beverly, USA). RIPA lysis buffer was purchased from Solarbio Life Sciences (Beijing, China). Briefly, cell samples or tissue samples were collected and lysed using RIPA reagent supplemented with phenylmethylsulfonyl fluoride (Roche) and a protease inhibitor cocktail (Roche, Pleasanton, CA, USA). The concentrations of all protein samples were detected using a BCA Protein assay kit (Beyotime). Equal amounts of protein extracts were added to gel wells and separated by 10% SDS-polyacrylamide gel electrophoresis gels. After electrophoresis and transferring protein bands to polyvinylidene fluoride membranes, the membranes were blocked for 1 h with 5% non-fat milk dissolved in Tris-buffered saline and incubated with primary antibodies at 4 °C for more than 12 h. The HRP-conjugated secondary antibodies were used and antigen–antibody complexes were tested by enhanced chemiluminescence system (Pierce, Rockford, IL, USA) according to the directions of the manufacturer.

### Tumor xenografts and tail vein injection experiments

All BALB/c nude mice (4 weeks old, female) were maintained under pathogen-free conditions and all procedures for the mouse experiments were approved by the Animal Care Committee of Qingdao University. For the tumor xenografts experiments, BGC-823 cells (1 × 10^6^, 100 µl) infected with lentivirus (polybrene, 8 μg/ml) were subcutaneously injected into mice (*n* = 6/group). Tumor growth was examined every 3 days, and tumor volumes were calculated using the equation: length × width^2^ × 0.5. After 29 days post injection, mice were euthanized, and the tumors were excised, photographed, and tissue sections were obtained for qRT-PCR detection or immunoblotting or IHC staining. For the tail vein injection experiment, BGC-823 cells (1 × 10^6^, 100 µl) were injected into the tail veins of six mice, which were euthanized 4 weeks after injection. The lungs were excised, photographed, and visible tumor nodules on the lung surface were counted, then fixed for later HE staining.

### IHC staining

IHC staining was performed on the paraffin-embedded tumor tissues from nude mice. The avidin-biotin-peroxidase method was adopted to determine the location and relative expression level of the target proteins. The primary antibodies of CDK4 and ki-67 were used at a dilution of 1:200. Sections were visualized under a microscope at ×400 (Olympus, Japan).

### Statistical analysis

All statistical analyses were performed with SPSS 20.0 (SPSS, Chicago, USA). Data were represented as mean ± standard deviation based on at least three repeats. Group difference was assessed using paired *t* test, two-sample *t* test, or one-way analysis of variance followed by post hoc Dunnett’s multiple comparisons test. Association was identified by Pearson correlation analysis. *p* < 0.05 was considered as statistically significant.

## Electronic supplementary material

Supplementary Figure Legends

Supplementary Fig. S1

Supplementary Fig. S2

Supplementary Fig. S3

Supplementary Fig. S4

Supplementary Fig. S5

Supplementary Fig. S6

Supplementary Fig. S7

Supplementary Table 1

Supplementary Table 2
