# Supplementary material for: Long noncoding RNA gastric cancer-related lncRNA1 mediates gastric malignancy through miRNA-885-3p and cyclin-dependent kinase 4
Source: Cell Death Dis. 2018 May 22;9(6):607. doi: 10.1038/s41419-018-0643-5 (PMC5964145; doi:10.1038/s41419-018-0643-5)
Supplement: Supplementary file 1 — Supplementary Figure Legends [file 41419_2018_643_MOESM1_ESM.docx]

**SUPPLEMENTARY FIGURE LEGENDS**

**Supplementary Fig. S1 Effects of GCRL1 on GC cells apoptosis *in* *vitro*.**

(A-B) Apoptosis analysis of si-GCRL1s-transfected BGC-823 cells after Annexin V/PI staining by cell sorting through flow cytometry and results were shown as the percentages of apoptotic cells. (C-D) Apoptosis analysis of MGC-803 cells with GCRL1 overexpression after Annexin V/PI staining by cell sorting through flow cytometry and results were shown as the percentages of apoptotic cells. Data are expressed as mean ± SD, * *p* < 0.05 compared to si-nc (B) or pc3.1 (D), n = 3.

**Supplementary Fig. S2 Expression of Lv-shGCRL1s in BGC-823 cells.**

(A) Bright field image (left) and fluorescent image (right) of lentivirus-infected BGC-823 cells 7 days after infection with Lv-shGCRL1s. (scale bars = 50 *μ*m) (B) Expression levels of GCRL1 were determined by qRT-PCR in BGC-823 cells infected with Lv-shGCRL1s. Data are expressed as mean ± SD, * *p* < 0.05 compared to Lv-nc-infected BGC-823 cells.

**Supplementary Fig. S3 Expression levels of miR-185-3p and miR-1250-5p in gastric cancer tissues and cell lines.**

(A) Expression levels of miR-185-3p and miR-1250-3p in paired GC tissues, n = 26. (B) Expression levels of miR-185-3p and miR-1250-3p in gastric cancer cell lines (SGC-7901, MGC-803, BGC-823 and AGS) and normal gastric epithelium cell line GES-1 (n = 3). Data are expressed as mean ± SD of fold change, * *p* < 0.05 compared to adjacent non-tumor tissues (A) or GES-1 (B).

**Supplementary Fig. S4 Construction of GCRL1-BS-WT/Mut reporter vector.**

The wild type GCRL1 fragment containing the binding site of miR-885-3p (GCRL1-BS-WT) or its mutant (GCRL1-BS-Mut) was cloned into pGL3-control reporter plasmid as indicated. The mutant sites were labeled in lowercase letters and with underlines.

**Supplementary Fig. S5 Inhibition of miR-885-3p enhances GC cells proliferation, migration and invasion *in vitro*.**

(A) Expression levels of miR-885-3p were determined by qRT-PCR in MGC-803 cells transfected with AntagomiR-885 or AntagomiR-nc, which were normalized to human U6. n = 3 (B) EdU incorporation assay assessing the proliferation of MGC-803 cells treated with AntagomiR-885 or AntagomiR-nc and representative images were shown (scale bars = 50 *μ*m). (C) Bar graphs of cell multiplication to Fig.S5B upon EdU incorporation assay and results were shown as the percentage of EdU positive cells to hoechst positive cells. n = 5 (D) Transwell assay indicating the mobility of MGC-803 cells treated with AntagomiR-885 or AntagomiR-nc and representative images were shown. (scale bars = 100 *μ*m) (E) Bar graphs of cell mobility to Fig. S5D upon transwell assay and results were shown as the number of migrated or invaded cells per field. n = 5. Data are expressed as mean ± SD, * *p* < 0.05 compared to AntagomiR-nc (A, C, E).

**Supplementary Fig. S6 Expression of miR-885-3p in Lv-miR-885-3p treated BGC-823 cells.**

(A) Bright field image (left) and fluorescent image (right) of lentivirus-infected BGC-823 cells 7 days after infection with Lv-miR-885-3p or Lv-nc. (scale bars = 50 *μ*m) (B) Expression levels of GCRL1 were determined by qRT-PCR in BGC-823 cells infected with Lv-miR-885-3 or Lv-nc. n = 3. Data are expressed as mean ± SD, * *p* < 0.05 compared to Lv-nc-infected BGC-823 cells.

**Supplementary Fig. S7 Effects of CDK4 on GC cells proliferation, migration and invasion *in vitro*.**

(A) Expression levels of CDK4 were examined by western blotting in BGC-823 cells transfected with si-CDK4 or si-nc, with β-actin as controls. (B) EdU incorporation assay assessing the cell proliferation of BGC-823 treated with si-CDK4 and representative images were shown (scale bars = 50 *μ*m). (C) Bar graphs of cell multiplication to Fig.S7B upon EdU incorporation assay and results were shown as the ratio of EdU positive cells to hoechst positive cells. (D) Bar graphs showed the migration and invasion ability of BGC-823 cells with CDK4 knockdown in transwell assay and results were shown as the number of migrated or invaded cells per field. n = 5. (E) Expression levels of CDK4 were examined by western blotting in MGC-803 cells with CDK4 overexpression, with β-actin as controls. (F) EdU incorporation assay assessing the cell proliferation of MGC-803 with CDK4 overexpression and representative images were shown (scale bars = 50 *μ*m). (G) Bar graphs of cell multiplication to Fig.S7F upon EdU incorporation assay and results were shown as the ratio of EdU positive cells to hoechst positive cells. n = 5. (H) Bar graphs showed the migration and invasion ability of MGC-803 cells after CDK4 overexpression in transwell assay and results were shown as the number of migrated or invaded cells per field. n = 5. Data are expressed as mean ± SD, * *p* < 0.05 compared to si-nc (C, D) or pc3.1 (G, H).
