# Supplementary figures and images for: Long noncoding RNA gastric cancer-related lncRNA1 mediates gastric malignancy through miRNA-885-3p and cyclin-dependent kinase 4
Source: Cell Death Dis. 2018 May 22;9(6):607. doi: 10.1038/s41419-018-0643-5 (PMC5964145; doi:10.1038/s41419-018-0643-5)

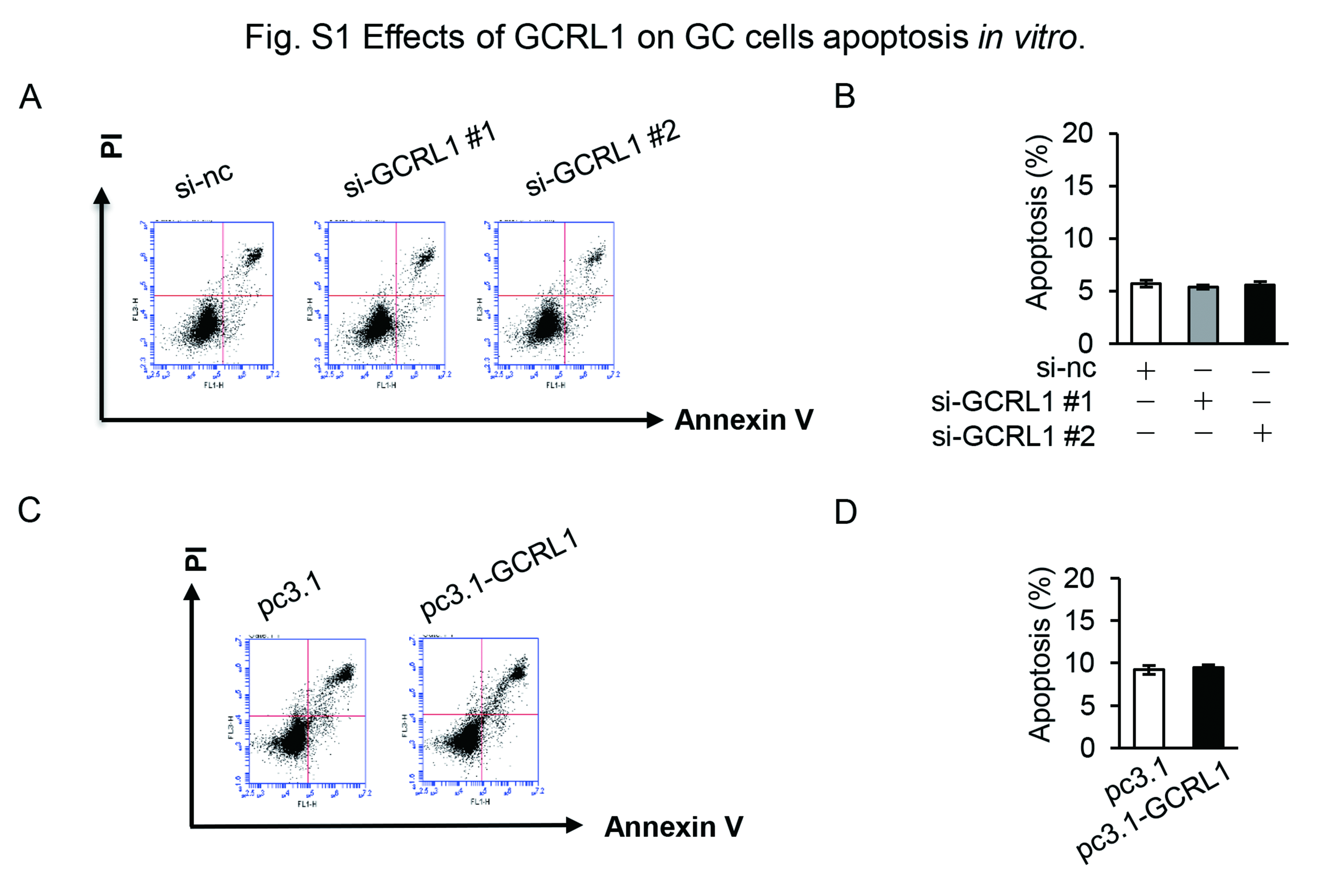

Supplement: Supplementary file 2 — Supplementary Fig. S1 [file 41419_2018_643_MOESM2_ESM.tif]

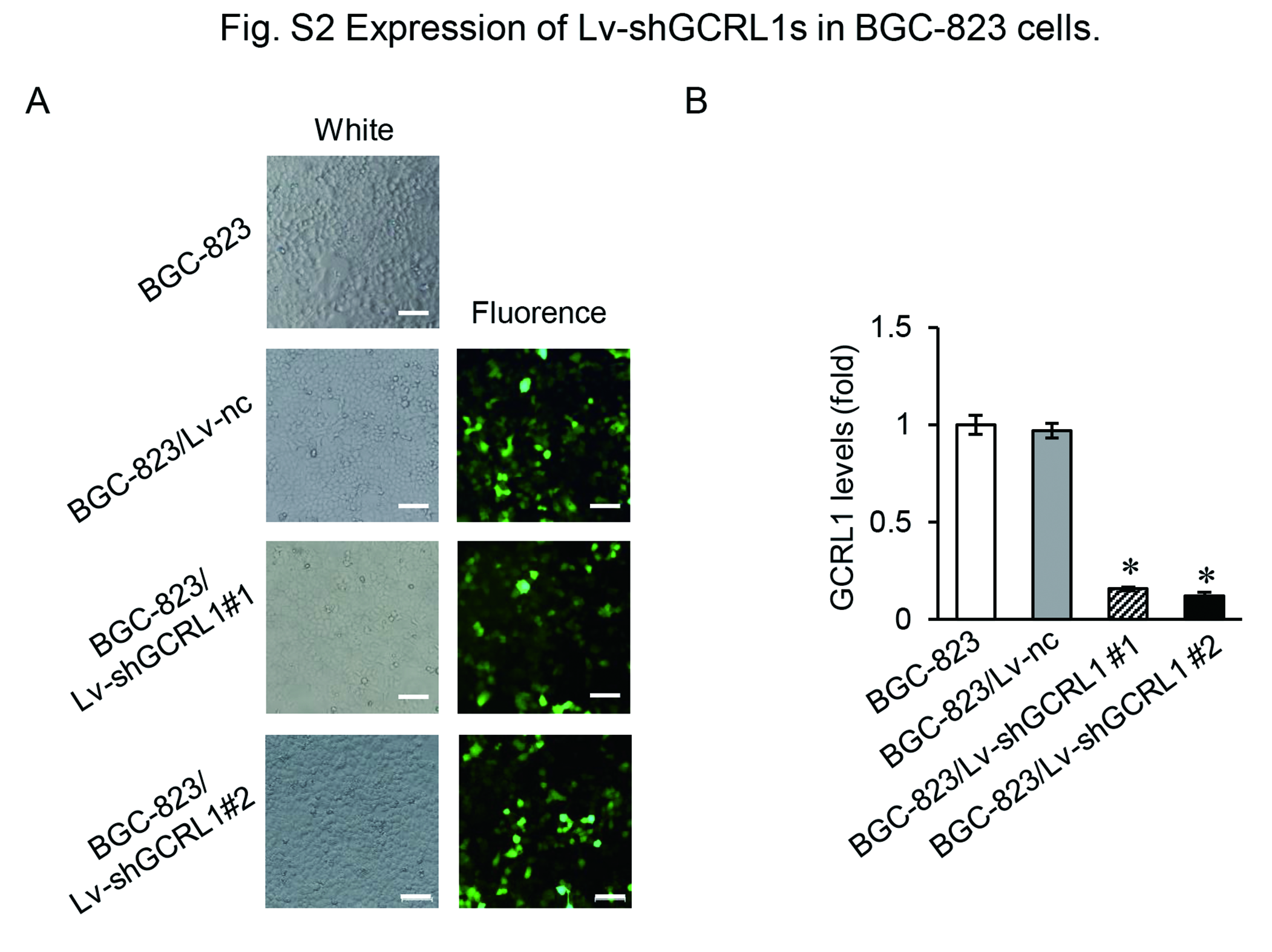

Supplement: Supplementary file 3 — Supplementary Fig. S2 [file 41419_2018_643_MOESM3_ESM.tif]

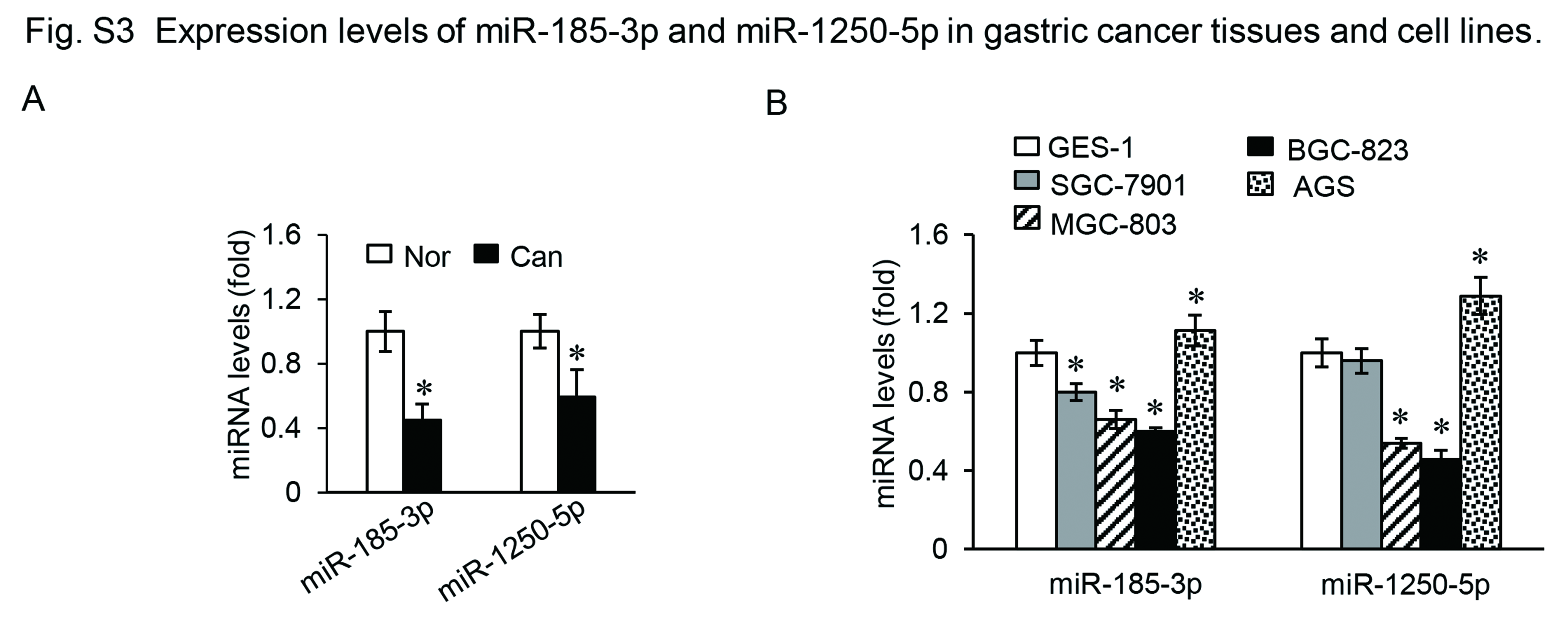

Supplement: Supplementary file 4 — Supplementary Fig. S3 [file 41419_2018_643_MOESM4_ESM.tif]

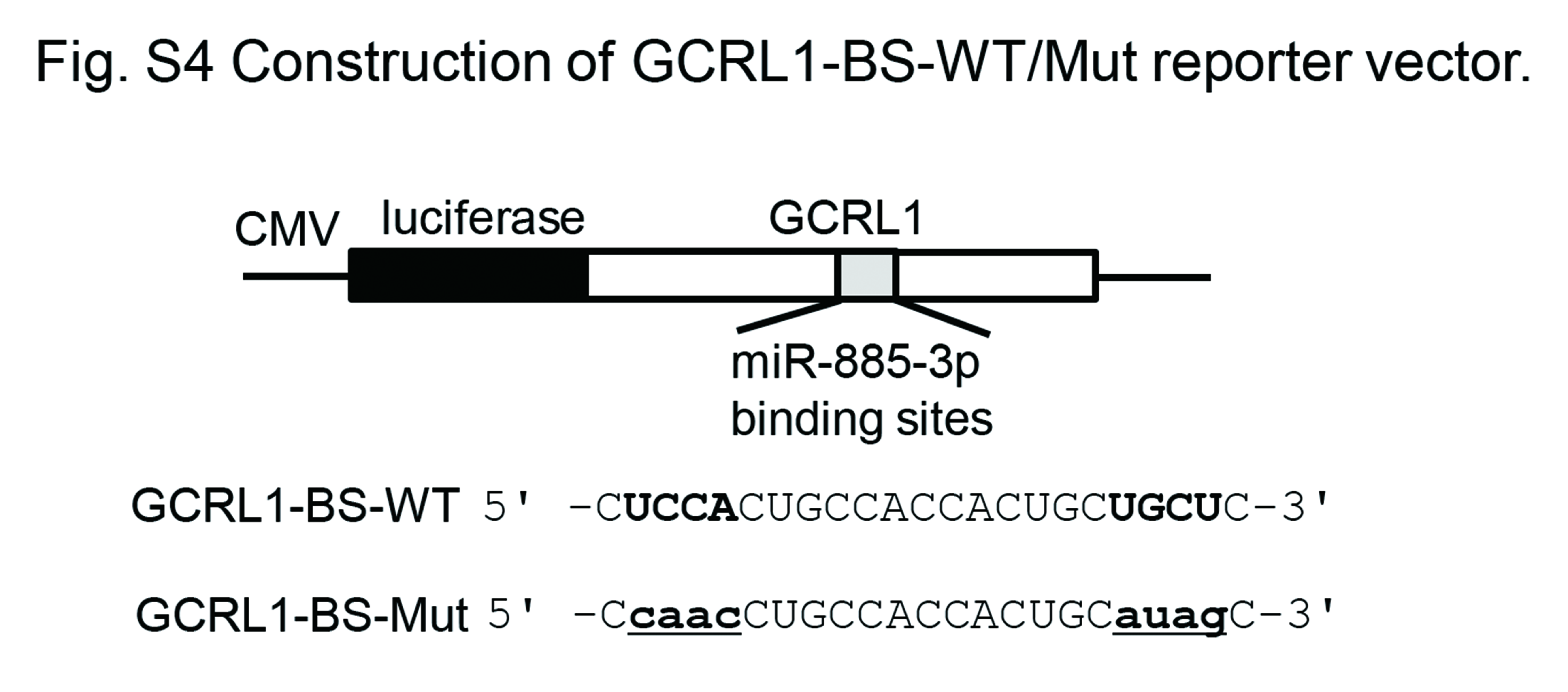

Supplement: Supplementary file 5 — Supplementary Fig. S4 [file 41419_2018_643_MOESM5_ESM.tif]

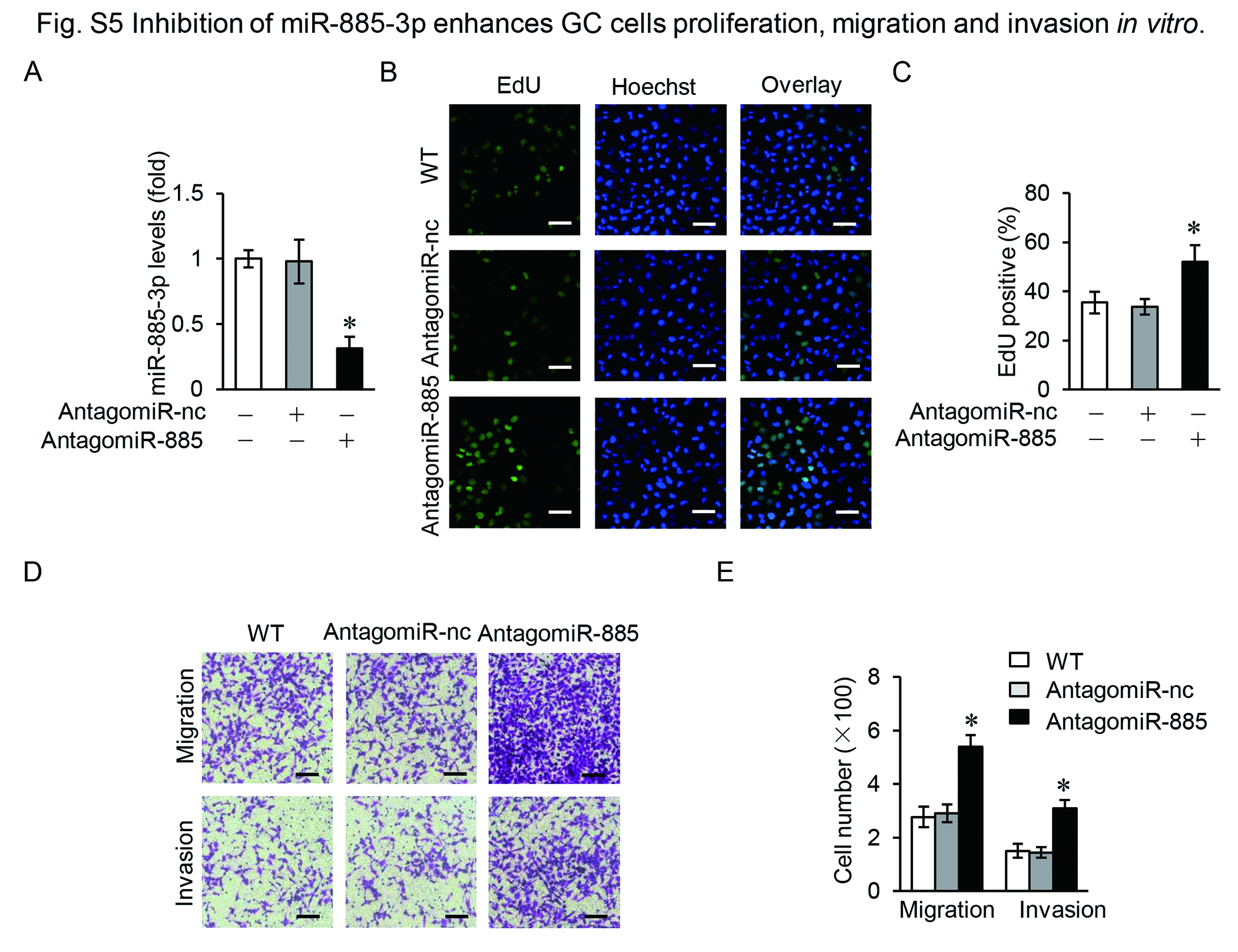

Supplement: Supplementary file 6 — Supplementary Fig. S5 [file 41419_2018_643_MOESM6_ESM.tif]

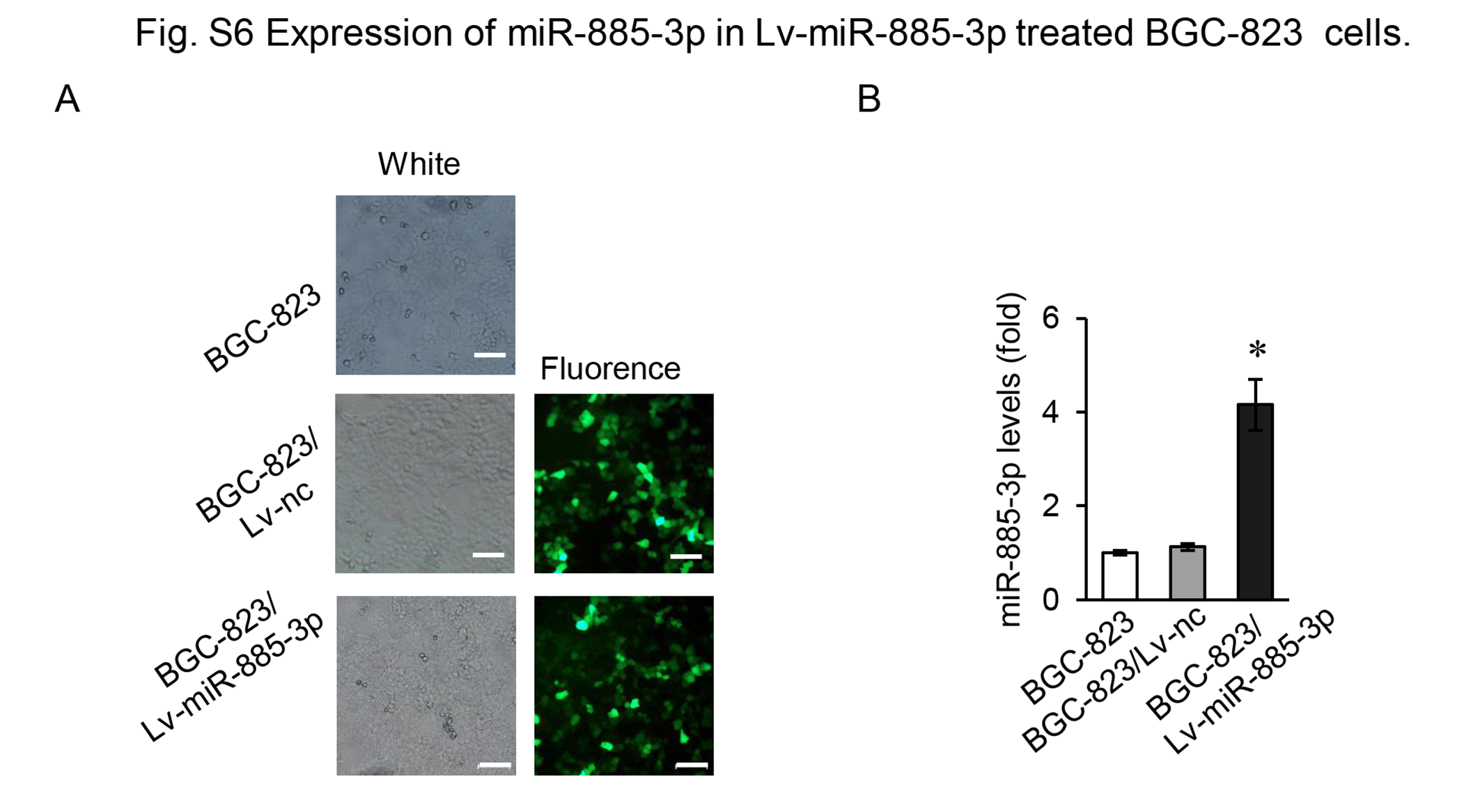

Supplement: Supplementary file 7 — Supplementary Fig. S6 [file 41419_2018_643_MOESM7_ESM.tif]

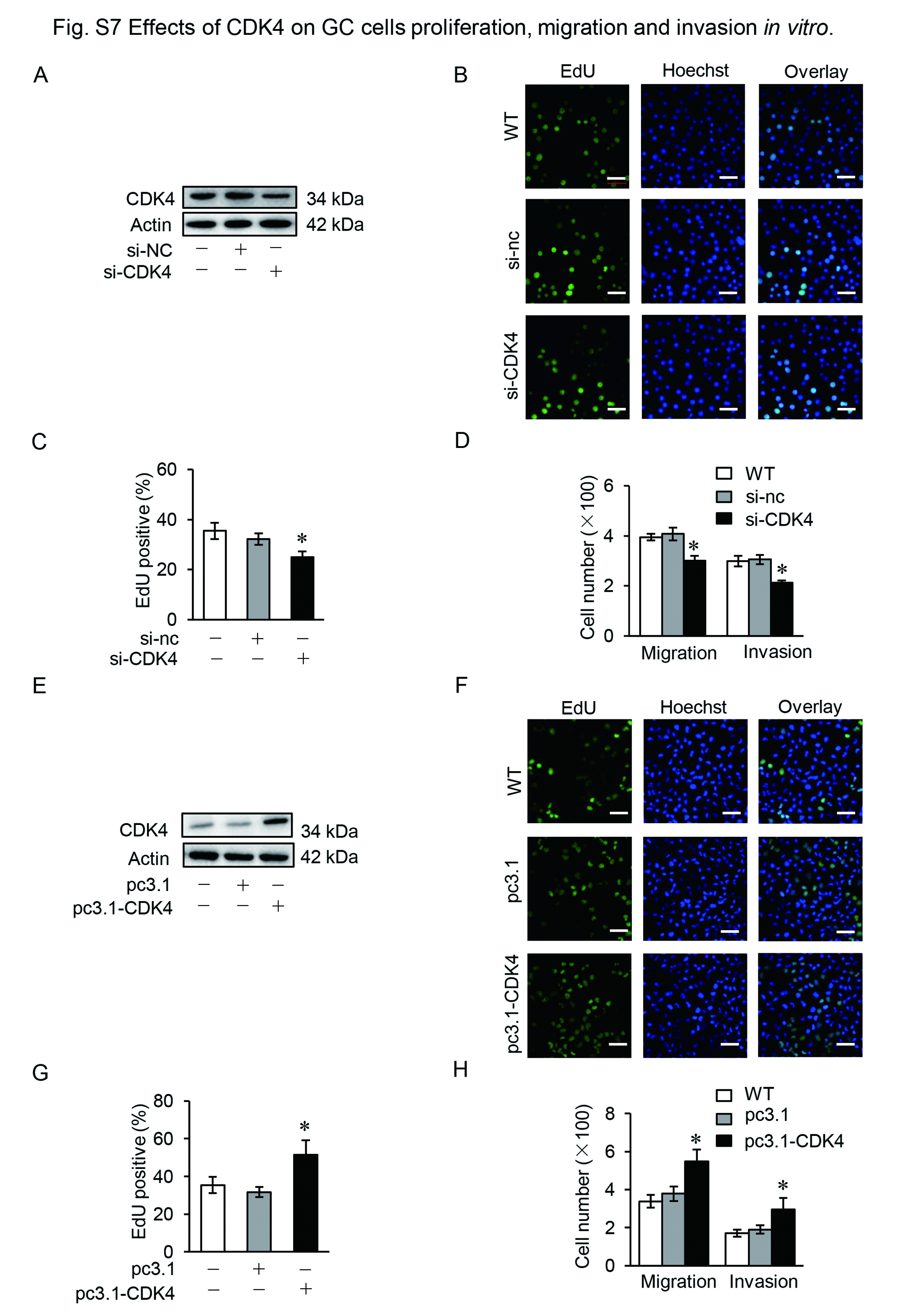

Supplement: Supplementary file 8 — Supplementary Fig. S7 [file 41419_2018_643_MOESM8_ESM.tif]
