# Supplementary material for: Long noncoding RNA gastric cancer-related lncRNA1 mediates gastric malignancy through miRNA-885-3p and cyclin-dependent kinase 4
Source: Cell Death Dis. 2018 May 22;9(6):607. doi: 10.1038/s41419-018-0643-5 (PMC5964145; doi:10.1038/s41419-018-0643-5)
Supplement: Supplementary file 9 — Supplementary Table 1 [file 41419_2018_643_MOESM9_ESM.docx]

**Supplementary Table 1. Characteristics of patients with gastric cancer**

| **Clinicopathologic features** | **n** |
| --- | --- |
| **Age (years)** |  |
| >55 | 17 |
| ≤55 | 9 |
| **Gender** |  |
| Female | 5 |
| Male | 21 |
| **Tumor size (cm)** |  |
| <4 | 9 |
| ≥4 | 17 |
| **Tumor invasion depth (T)** |  |
| T1 | 4 |
| T2 | 1 |
| T3 | 5 |
| T4 | 16 |
| **Lymph node metastasis (N)** |  |
| N0 | 5 |
| N1 | 7 |
| N2 | 9 |
| N3 or above | 5 |
| **Distant metastasis (M)** |  |
| M0 | 21 |
| M1 | 5 |
| **TNM stage** |  |
| Ⅰ-Ⅱ | 5 |
| Ⅲ-Ⅳ | 21 |
| **Lauren classification** |  |
| Intestinal | 15 |
| Diffuse | 10 |
| Mixed | 1 |
