# Supplementary material for: Long noncoding RNA gastric cancer-related lncRNA1 mediates gastric malignancy through miRNA-885-3p and cyclin-dependent kinase 4
Source: Cell Death Dis. 2018 May 22;9(6):607. doi: 10.1038/s41419-018-0643-5 (PMC5964145; doi:10.1038/s41419-018-0643-5)
Supplement: Supplementary file 10 — Supplementary Table 2 [file 41419_2018_643_MOESM10_ESM.docx]

**Supplementary Table 2. Sequence and primer information**

| **Sequence name** | **core sequence(5'---3')** | |
| --- | --- | --- |
| si-*GCRL1*#1 | | GCTGGCACAGAAGTCTCTT |
| si-*GCRL1*#2 | | GGAGAATAAACCCTCGGAT |
| si-CDK4 | | GGAGTGTTGGCTGTATCTT |
| **Primers for qPCR** | | **sequence(5'---3')** |
| *GCRL1*-F | | GATCCTAGAGGAAAGTGGCAAG |
| *GCRL1*-R | | GGGTGTACAGCAGTGAACAA |
| \| *GCRL1*-F2 \| \| --- \| \| *GCRL1*-R2 \| | | AATGTCACCATCGCCCAGGT  CTCTGGCTGAGTGTGGAGAG |
| \| *GCRL1*-F3 \| \| --- \| \| *GCRL1*-R3 \| | | GAAAGTGGCAAGGCCAGGA  CAGCAGAGATGAGCAGCAGT |
| hCDK4-F | | TCGTGAGGTGGCTTTACTGAGGC |
| hCDK4-R | | CTTAGGTCCTGGTCTACATGCTC |
| miR-185-3p-F | | AGGGGCTGGCTTTCCT |
| miR-885-3p-F | | AGGCAGCGGGGTGTAG |
| miR-1250-5p-F | | ATAAACGGTGCTGGATGTG |
| URP(reverse) | | TGGTGTCGTGGAGTCG |
| human U6-F | | CTCGCTTCGGCAGCACA |
| human U6-R | | AACGCTTCACGAATTTGCGT |
| human GAPDH-F | | GGACTCATGACCACAGTCCATGCC |
| human GAPDH-R | | TCAGGGATGACCTTGCCCACAG |
| **Primers for RT reaction** | | **sequence(5'---3')** |
| miR-185-3p-R | | CTCAACTGGTGTCGTGGAGTCGGCAATTCAGTTGAGGACCAGAG |
| miR-885-3p-R | | CTCAACTGGTGTCGTGGAGTCGGCAATTCAGTTGAGTATCCACT |
| miR-1250-5p-R | | CTCAACTGGTGTCGTGGAGTCGGCAATTCAGTTGAGAAAGGCCA |
| **Primers for pull-down assay** | | **sequence(5'---3')** |
| Bio-nc | | UUCUCCGAACGUGUCACGUUU |
| Bio-885 | | AGGCAGCGGGGUGUAGUGGAUA |
| Bio-885 mut | | AUACGCGGGGUGUAGCUACUA |
| **Primers for plasmid construction** | | **sequence(5'---3')** |
| *GCRL1*-BS-WT-F | | ATCCCTTCCTTCTGCCTCCACTGCCACCACTGCTGCTCATCTCTGCTGGCA |
| *GCRL1*-BS-WT-R | | CTAGTGCCAGCAGAGATGAGCAGCAGTGGTGGCAGTGGAGGCAGAAGGAAGGGAT |
| *GCRL1*-BS-Mut-F | | GCCAGCAGAGATGTTACTTAGTGGTGGCGTGTCCGGCAGAAGGAAGG |
| *GCRL1*-BS-Mut-R | | CTAGTGCCAGCAGAGATGTTACTTAGTGGTGGCGTGTCCGGCAGAAGGAAGGGAT |
| LV-*GCRL1*-shRNA#2F | | CCGGGGAGAATAAACCCTCGGATCTCGAGATCCGAGGGTTTATTCTCCTTTTTG |
| LV-*GCRL1*-shRNA#2R | | AATTCAAAAAGGAGAATAAACCCTCGGATCTCGAGATCCGAGGGTTTATTCTCC |
| CDK4-pF | | CGGAATTCCACCTCCTGTCCGCCCCTCA |
| CDK4-pR | | CGGGATCCCTCCGGATTACCTTCATCCT |
| LV-885-F | | CCGGAGGCAGCGGGGTGTAGTGGATACTCGAGTATCCACTACACCCCGCTGCCTTTTTTG |
| LV-885-R | | AATTCAAAAAAGGCAGCGGGGTGTAGTGGATACTCGAGTATCCACTACACCCCGCTGCCT |
